# A freely precessing magnetar following an X-ray outburst

**DOI:** 10.1038/s41550-024-02226-7

**Published:** 2024-04-08

**Authors:** Gregory Desvignes, Patrick Weltevrede, Yong Gao, David Ian Jones, Michael Kramer, Manisha Caleb, Ramesh Karuppusamy, Lina Levin, Kuo Liu, Andrew G. Lyne, Lijing Shao, Ben Stappers, Jérôme Pétri

**Affiliations:** 1 Max-Planck-Institut für Radioastronomie, Bonn, Germany; 2 Laboratoire d’Études Spatiales et d’Instrumentation en Astrophysique, Observatoire de Paris, Université Paris-Sciences-et-Lettres, Centre National de la Recherche Scientifique, Sorbonne Université, Université de Paris, Meudon, France; 3 Jodrell Bank Centre for Astrophysics, The University of Manchester, Manchester, UK; 4 Department of Astronomy, School of Physics, Peking University, Beijing, China; 5 Kavli Institute for Astronomy and Astrophysics, Peking University, Beijing, China; 6 Mathematical Sciences and STAG Research Centre, University of Southampton, Southampton, UK; 7 Sydney Institute for Astronomy, School of Physics, The University of Sydney, Sydney, New South Wales Australia; 8 ASTRO3D: ARC Centre of Excellence for All-sky Astrophysics in 3D, Canberra, Australian Capital Territory Australia; 9 National Astronomical Observatories, Chinese Academy of Sciences, Beijing, China; 10 Université de Strasbourg, CNRS, Observatoire astronomique de Strasbourg, UMR 7550, Strasbourg, France

**Keywords:** Compact astrophysical objects, Transient astrophysical phenomena

## Abstract

Magnetars—highly magnetized neutron stars—are thought to be the most likely progenitors for fast radio bursts (FRBs). Freely precessing magnetars are further invoked to explain the repeating FRBs. We report here on new high-cadence radio observations of the magnetar XTE J1810–197 recorded shortly after an X-ray outburst. We interpret the polarization variations of the magnetar radio emission as evidence for the magnetar undergoing free precession following the outburst while its magnetosphere slowly untwists. The observations of precession being damped on a timescale of months argue against the scenario of freely precessing magnetars as the origin of repeating FRBs. Using free-precession models based on relaxing ellipticity with a decay of the wobble angle, we find the magnetar ellipticity to be in good agreement with theoretical predictions from nuclear physics. Our precise measurement of the magnetar’s geometry can also further help in refining the modelling of X-ray light curves and constrain the star’s compactness.

## Abstract (web summary)

A radio-loud magnetar, XTE J1810–197, has been observed to precess shortly after an X-ray outburst. The precession decayed over the subsequent few months, which probably rules out freely precessing magnetars as the source of the fast radio bursts.

## Main

Magnetars are rare, typically slowly spinning neutron stars whose emission is thought to be powered by the decay of their large (≥10^12^ G) magnetic fields^1^ in opposition to normal rotation-powered pulsars. They occasionally undergo bright X-ray outburst phases, supposedly originating from a sudden quake in the star’s crust, resulting in a twisted magnetosphere that fuels the outburst^2^. Out of the 24 confirmed magnetars currently known, only 6 have been shown to emit in the radio band^3^.

The discovery of a bright fast radio burst (FRB) from the galactic magnetar SGR 1935+2154 recently gave strong credence to radio magnetars being the progenitors of at least some extra galactic FRBs^4^ (see, for example, ref. ^5^ for a review on FRBs). Models of freely precessing magnetars were then put forward to explain the periodicities in the activity observed in the train of pulses from some repeating FRBs^6,7^.

The first recorded X-ray outburst of the magnetar XTE J1810–197 (position $1{8}^{{\mathrm{h}}} 0{9}^{{\mathrm{min}}} 51.0{7}^{{\mathrm{s}}}-1{9}^{\circ } 4{3}^{{\prime} } 51.{8}^{{\prime}{\prime} }$, J2000) happened around late 2002 (ref. ^8^) and bright radio pulsations were detected 3 years later^9^ with a periodicity of *P*_s_ = 5.54 s. The radio emission slowly decayed with time until it ceased to be detected in late 2008 (ref. ^10^). Radio pulsations from XTE J1810–197 were again detected on 8 December 2018 (Modified Julian Date (MJD) 58,460.6)^11^, following a second X-ray outburst known to have occurred between 20 and 26 November 2018 (ref. ^12^).

We present a set of 62 polarimetric observations of XTE J1810–197 recorded with the Lovell and Effelsberg radio telescopes at 1.5 and 6 GHz, respectively, between 8 December 2018 and 18 June 2020 (MJD 59,018). These observations provide calibrated polarimetric pulse profiles with almost daily cadence during the first month of monitoring. An overview of the observations and data processing can be found in the Methods section.

The polarimetric pulse profiles exhibit a high degree of linear polarization *L* at both frequencies, consistent with previous observations of this magnetar^13–15^. Thanks to our observing cadence, we noticed rapid and systematic changes of the position angle (PA) of *L* with time (Supplementary Figs. 1–7), including two reversals in the sign of the gradient of the PA, first between MJDs 58,464–58,466, also previously reported in ref. ^15^, and a second one between MJDs 58,551–58,589.

The standard model to interpret the polarization of a pulsar’s profile is the rotating vector model (RVM)^16^. Assuming a dipolar field geometry, it describes the observed PA sweep of the polarized radio emission as it crosses our line of sight. Under this theory, the exact shape of the PA sweep is determined by the viewing geometry, that is the angle between the pulsar spin axis and the pulsar-observer line of sight *ζ* = *α* + *β* where *α* is the magnetic inclination angle and *β* is the angle between the magnetic axis and our line of sight at its closest approach^17^, hereafter referred to as the impact parameter. Although deviations from the RVM can be observed (due to, for example, propagation effects throughout the pulsar magnetosphere), the validity of the geometrical nature of the RVM has been demonstrated, at least for rotation-powered pulsars^18^.

An extension to the RVM for the suggested twisted magnetic fields of magnetars^2,19,20^ predicts that the untwisting of the magnetic field can produce a vertical shift in PA, and to a lesser extent a horizontal shift in rotational phase, of the PA sweep while retaining the same PA gradient under the pulse. The off-centred dipole model for polarization^21^ does predict some small changes in the gradient of the PA but none of the theories can account for the reversal in the sign of the PA gradient without a change in the viewing geometry. Furthermore, at the radio emission height of tens of thousands of km above the magnetar’s surface, inferred from our polarization data (Methods), multipolar magnetic fields are unlikely to be dominant over the dipolar magnetic field. The sudden apparent change in the gradient of the PA of the magnetar Swift J1818.0–1607 on MJD 59,062 (ref. ^22^), together with a depolarization of the linear polarization, can be explained by an apparent orthogonal polarization mode^23^. Precession is therefore the only known physical process that could cause the observed systematic variations in the PA and its PA gradient sign reversal. In this work, we consider both forced precession and free precession.

In the case of forced precession, the direction of the neutron star (NS) spin vector changes significantly, due to, for example, its motion in a curved spacetime caused by a massive companion star. In this case, *α* remains fixed with time and *β* is modulated by the relativistic spin-precession period as predicted by Einstein’s theory of General Relativity^18,24^.

**Fig. 1:**
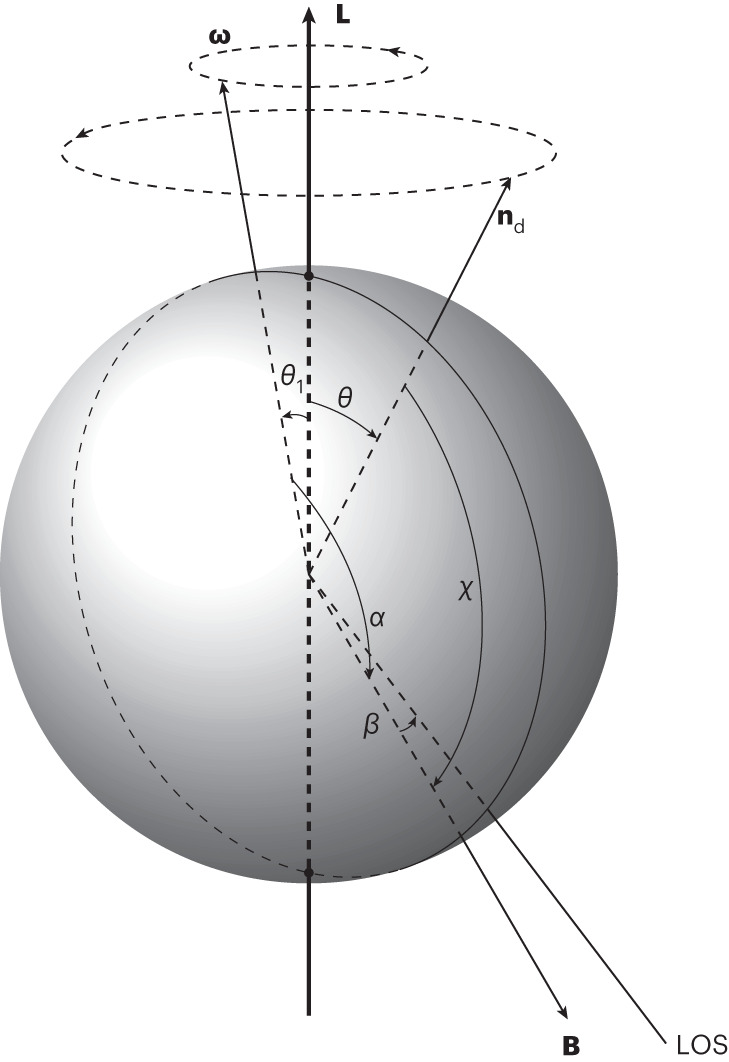
Geometry of a freely precessing biaxial neutron star in the inertial frame. The NS spin vector **ω** of period *P*_s_ rotates around the angular momentum vector **L** with a very small angle *θ*_1_. The deformation axis **n**_d_ is misaligned with respect to **L** by *θ*. The NS magnetic axis **B** rotates around **n**_d_ at an angle *χ* with the free-precession period ${P}_{{{{\rm{f}}}}} \approx \frac{{P}_{{{{\rm{s}}}}}}{\epsilon \cos \theta }$ where *ϵ* is the ellipticity of the NS. **L**, **ω** and **n**_d_ always lie in the same plane. As the NS precesses, the magnetic inclination angle *α* and *β*, the closest approach between the NS magnetic axis and our line of sight (LOS), varies with time while *ζ* = *α* + *β* is assumed to be constant.

In contrast, in the case of free precession (see, for example, ref. ^25^), precession originates from the aspherical deformation of the NS expressed as its ellipticity *ϵ*. The angle between this deformation axis and the angular momentum of the NS is defined as the wobble angle *θ*. The direction of the NS spin vector is assumed to be approximately fixed in space, that is *ζ* remains constant with time. The magnetic axis precesses around the symmetry axis misaligned with an angle *ξ*, causing periodic modulations in *α* and *β*. This precession occurs at the frequency ${\omega }_{{{{\rm{p}}}}}=\epsilon \omega \cos \theta$ where $\omega =\frac{2\uppi }{{P}_{{{{\rm{s}}}}}}$ is the NS angular frequency. Figure 1 illustrates the free-precession geometry.

Despite initial claims of detections of free precession in the pulsars B1642–03 and B1828–11 through the timing of their pulse arrival times^26,27^, these pulsars have since then been shown to exhibit magnetospheric mode changes causing the observed profile variation^28,29^, complicating the interpretation of their timing behaviour. Free precession has also been invoked to explain the pulse phase modulation with period <1 day observed in the hard X-ray emission of the magnetars 4U 0142+61 (ref. ^30^), 1E 1547.0–5408 (ref. ^31^) and SGR 1900+14 (ref. ^32^).

Although XTE J1810–197 is not known to orbit around a massive companion, we nonetheless attempted to compare the cases of forced and free precession. We performed a simultaneous fit of the RVM to all PA data from our 62 epochs, with a common *α* parameter between epochs in the case of forced precession. For the free-precession scenario, we replaced the common *α* parameter with a common *ζ* parameter. The remaining RVM parameters (*β* and two offset parameters, per epoch) are set free. Finally we applied the nested sampling tool POLYCHORD^33^ to explore the 189-dimension parameter space and compute the Bayesian log-evidence $\log {{{\mathcal{Z}}}}$ for each case. We take the 68% confidence levels on the one-dimensional marginalized posterior as our 1*σ* uncertainties (more details on the analysis can be found in Methods).

**Fig. 2:**
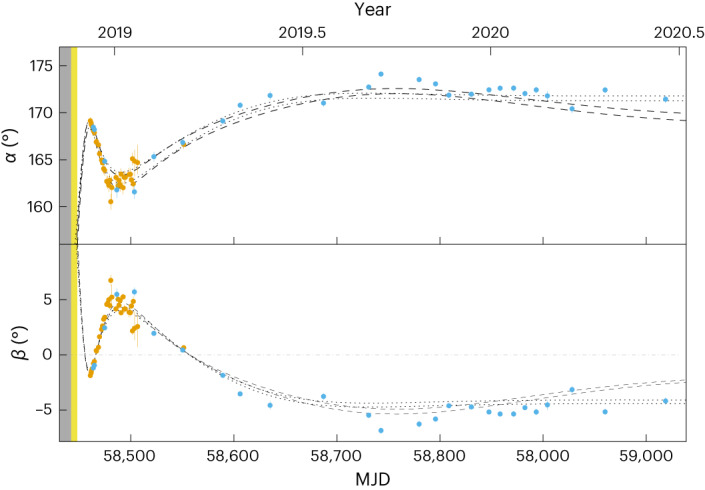
Temporal evolution of the geometry of XTE J1810–197. The top and bottom panels show the magnetic inclination angle *α* and the impact parameter *β*, respectively. Assuming the model independent free-precession scenario (constant *ζ* across all epochs), the orange and blue points represent the measurements from the Jodrell 1.5 GHz and Effelsberg 6 GHz data, respectively. We take the 95% confidence levels on the one-dimensional marginalized posterior as the uncertainties for the *α* and *β* measurements. The dot-dash line at *β* = 0° represents the crossing of the line of sight over the magnetic pole where the gradient of the PA is predicted to change sign. The period of time when the second X-ray outburst occurred is marked yellow. The dashed and dotted lines delimit the 95% confidence levels on *α* and *β* for the model of crust–core coupling with relaxing ellipticity and the phenomenological model of wobble angle decay with relaxing ellipticity model, respectively.

**Extended Data Fig. 1:**
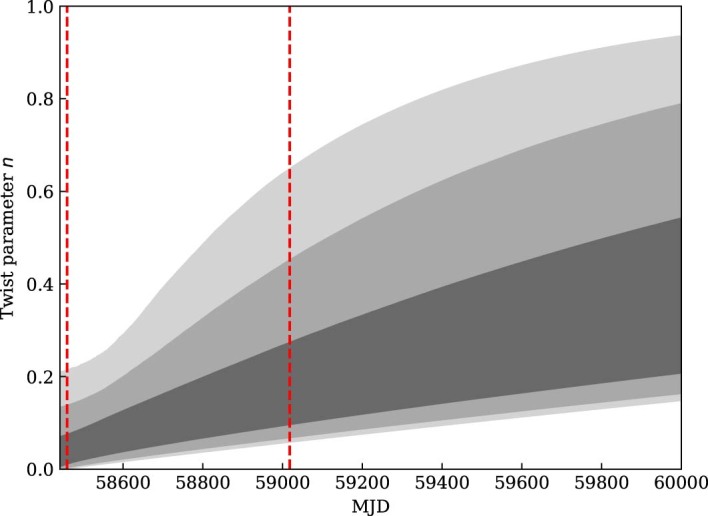
Predicted waveform for the temporal evolution of the twist parameter n included in the free precession modelling. The grey contours, from darker to lighter shade, show the 68%, 95% and 99.7% confidence levels for the value of n. The two vertical red dashed lines delimit the time window of our observations.

The log-Bayes factor, the difference in ‘log-evidence’ between the two models, is ~75 in favour of the free-precession model and therefore unarguably supports, as expected, free precession against forced precession as the origin of the observed PA changes. When including the effects of a decaying, eastward-twisted magnetic field (Methods), the log-Bayes factor increases to ∼244 in favour of the free-precession model. This analysis gives *ζ* = 167.26° ± 0.22° and we show in Fig. 2 the temporal variations of *α* and *β* according to this model, with 160° < *α* < 175° and ∣*β*∣ < 8°. From Fig. 2 we can see that the rapid rate of change of *α* and *β* (~0.4° day^−1^ at its observed maximum near MJD 58,470) is decreasing with time arguing for damped precession. We can constrain the time relaxation of the twisted magnetic field *τ*_t_ > 730 days (95% confidence level) with a large initial twist parameter *n*_0_ ≈ 0.04 at the time of the outburst (Extended Data Fig. 1). This evidence for the untwisting of the magnetosphere is consistent with the observation of the decrease in pulse width^34^ and the variable spin-down rate of the star and its delay after the outburst^35^.

**Extended Data Fig. 2:**
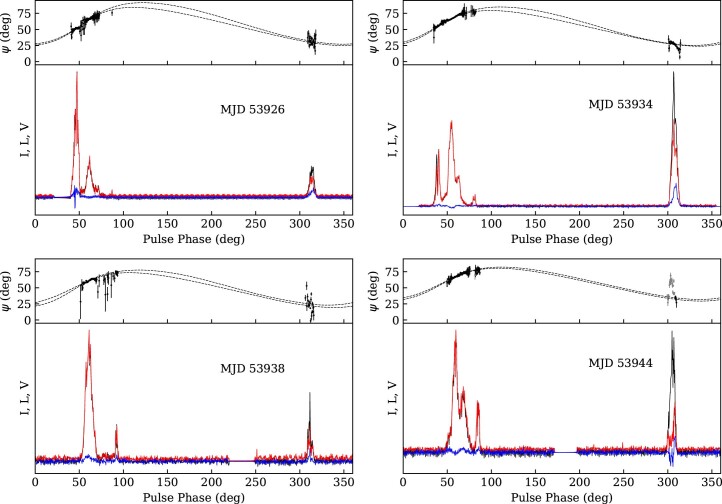
Polarimetric pulse profiles recorded at a frequency of 8.4 GHz with the Effelsberg radio telescope on four occasions during the summer of 2006. In the bottom part of each panel, the black, red and blue lines represent the total intensity I, linear L and circular V polarisation, respectively. In the top part, the black data points indicate the PA of the linear polarisation included in the modelling. The uncertainty in the PA is calculated given L and the off-pulse standard deviation of I^53^. The dotted lines delimit the 95% confidence levels on Ψ derived from the posterior distributions (seen in Extended Data Fig. 3) assuming the RVM.

**Extended Data Fig. 3:**
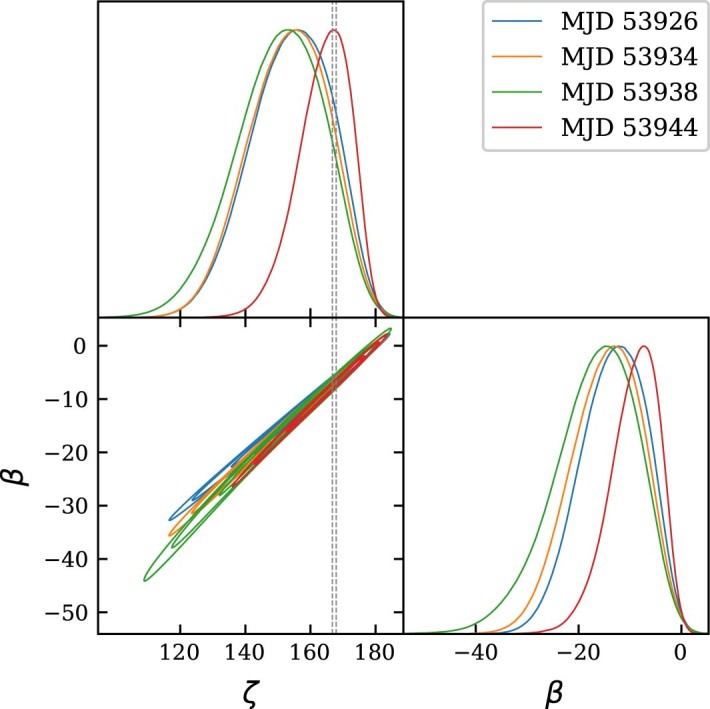
Posterior distributions for the *ζ* and *β* parameters from the RVM analysis of the Effelsberg archival data. The observations were recorded at 8.4 GHz on MJDs 53926, 53934, 53938 and 53944. The vertical dashed lines delimit the 95% confidence levels on *ζ* from our preferred free precession modelling of the 2018-2020 PA data with an eastward twisted magnetosphere.

In light of these new results, we reanalysed four epochs (Extended Data Figs. 2 and 3 and Methods) recorded with the Effelsberg radio telescope during the summer of 2006 (ref. ^13^), more than 3 years after the first recorded X-ray outburst, and applied the RVM to each of these epochs to revisit the viewing geometry of the magnetar in 2006. Taking the averaged *ζ* value, we find *ζ* = 155° ± 10°, indicating as expected a stable viewing angle between 2006 and 2018. These data could however not constrain the twisting of the magnetosphere. Assuming that free precession also occurred following the 2002 outburst of the magnetar (as we suggest for the 2018 outburst), it would probably have been damped by the time of the first radio detection in 2006, suggesting that *α* and *β* should have remained constant. Indeed, no variations in the shape of the PA were detected between 2006 and 2008 (ref. ^10^), confirming the stable geometry.

**Extended Data Fig. 4:**
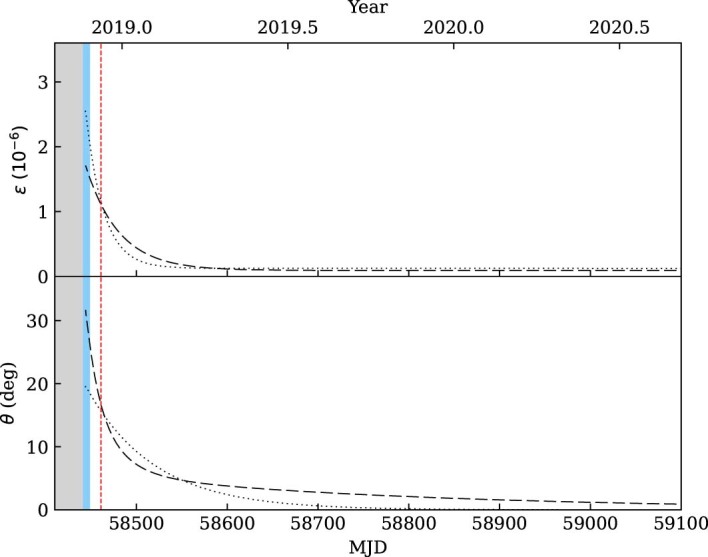
Temporal evolution of the ellipticity and wobble angle as predicted by the two best models of free precession. The top and bottom panels show the prediction for the ellipticity *ε* and wobble angle *θ*, respectively. Predictions (mean values) from the preferred model of relaxing ellipticity with frictional coupling are shown with dashed lines while predictions from the model of wobble angle decay with relaxing ellipticity to a non-zero value are shown with dotted lines. The light blue rectangle delimit the period of time when the second X-ray outburst occurred. The red vertical dashed line indicates the time of our first radio observation.

We also considered different models of biaxial free precession of a NS to interpret the observed PA variations, including relaxation of *ϵ* (to either a zero or non-zero value) and relaxation of *ϵ* combined with either a phenomenological or a frictional crust–core coupling model for the decay of *θ*. All models included the effects of a decaying and twisted magnetic field. As the geometry of XTE J1810–197 remained stable during 2006–2008, we assume here that free precession was set in motion at the time *T*_0_ of the 2018 X-ray outburst. We again used POLYCHORD to perform the model comparison (see Methods for more details on these models and our analysis). Our results (Extended Data Table 1) show with a Bayes factor of 45 that the preferred model (Table 1) is based on relaxing ellipticity combined with a phenomenological description for the decay of *θ*. The second preferred model is the crust–core coupling model with relaxation of *ϵ*. The predicted ellipticity *ϵ* (Extended Data Fig. 4) drops from a value of ∼(2.4 ± 0.1) × 10^−6^ and ~(1.7 ± 0.03) × 10^−6^ at *t* = *T*_0_ to a constant value of (1.25 ± 0.07) × 10^−7^ and (1.0 ± 0.02) × 10^−7^ about 5 months later for the preferred phenomenological and crust–core coupling models, respectively. The waveforms of the predicted geometry for these two models are shown in Fig. 2. Both predict a large positive *β* at the time of the X-ray outburst and a relatively stable negative *β* from 2020 onward. The discrepancy in *β* between 2006 and 2008 and the value predicted by our model at the epoch *T*_0_ could be attributed to either chaotic events at the time of the outburst that cannot be reproduced by our model or by the decay of ellipticity deviating from a simple exponential form.

**Table 1:** Parameter values for the two preferred models of free precession

Parameter	Value of model A	Value of model B
Viewing angle, *ζ* (deg)	169.07 ± 0.22	168.50_-0.21_^+0.26^
Initial wobble angle, *θ*_0_ (deg)	19.52 ± 0.38	30.52_-0.74_^+0.66^
Angle between the magnetic and symmetry axis, *χ* (deg)	173.34 ± 0.13	171.37_-0.16_^+0.20^
Initial phase of the precession, Φ_0_ (deg)	45_-4_^+5^	108 ± 3
Constant ellipticity of the NS, *ϵ*_0_	(1.24 ± 0.03) × 10^−7^	(9.17 ± 0.14) × 10^−8^
Initial ellipticity of the NS, *ϵ*_1_	(2.37 ± 0.05) × 10^−6^	(1.58 ± 0.03) × 10^−6^
Ellipticity relaxation timescale, *τ*_ϵ_ (days)	19.55 ± 0.35	36.43 ± 0.46
Wobble angle decay timescale, *τ*_θ_ (days)	74.30 ± 0.26	—
Frictional coupling timescale, *τ*_c_ (s)	—	2.49 ± 0.01
Ratio between the moment of inertia of the crust and the core, *κ*	—	<0.01
Start time of the precession, *T*_0_ (MJD)	58,444.5 ± 0.4	58,444.9 ± 0.5
Initial twist parameter, *n*_0_	0.040_-0.040_^+0.008^	0.062_-0.062_^+0.015^
Twisted magnetic field relaxation timescale, *τ*_t_ (days)	>1,500	>1,500

Model A denotes the phenomenological model of decaying wobble angle with relaxing ellipticity and model B denotes the model of frictional crust–core coupling with relaxing ellipticity. Both models include the effects of an decaying, eastward-twisted magnetic field.

The upper limit for the ellipticity of a NS derived from crustal elasticity^36–38^ is on the order of ${\epsilon }_{{{{\rm{ela}}}}} \approx 1{0}^{-6}\left(\frac{{\sigma }_{{{{\rm{br}}}}}}{0.1}\right)$. The parameter *σ*_br_ is the braking strain of the elastic crust. Molecular dynamics simulations for high pressure Coulomb crystals suggest that *σ*_br_ ≃ 0.1 (ref. ^39^). Strong internal magnetic fields *B* in the NS also create deformation with an ellipticity on the order of ${\epsilon }_{{{{\rm{mag}}}}} \approx 1{0}^{-6}{\left(\frac{B}{1{0}^{15}{{{\rm{G}}}}}\right)}^{2}$, which is a crude estimation but consistent with more rigorous calculations (for example, refs. ^40–43^). The maximum value of *ϵ* = 1.7 × 10^−6^ to ~2.4 × 10^−6^ at *t* = *T*_0_ depending on the model considered is consistent with the maximum ellipticity *ϵ*_ela_ ≈ 10^−6^ that a NS could sustain. Assuming the surface magnetic field strength *B*_surf_ ≈ 2.7 × 10^14^ G of XTE J1810–197 (ref. ^44^) is representative of the internal magnetic field, then *ϵ*_mag_ ≈ 7 × 10^−8^ is also broadly consistent with the constant *ϵ* ≈ 1 × 10^−7^ derived from the two preferred models. A possible interpretation is that the constant contribution to *ϵ* is sourced from *ϵ*_mag_ while the crustal strain causing the decaying contribution to *ϵ* relaxed in the first 3 months after the outburst.

XTE J1810–197 is not the only magnetar observed in radio prior and after an X-ray outburst. PSR J1622–4950 was first discovered by the High Time Resolution Universe South survey in 2009 (ref. ^45^) and monitored in radio until its emission ceased to be detected in 2014. Radio emission from this source was again detected on 5 April 2017, with an X-ray outburst happening two weeks before the radio redetection. However, the first polarimetric pulse profiles used for that study were recorded about 5 months after the outburst. A comparison of the polarimetric pulse profiles pre and post outburst did not reveal any large changes in the magnetar’s viewing geometry^46^. We can speculate that either the outburst of PSR J1622–4950 did not excite the free precession or the precession damped on a timescale similar to or shorter than that of XTE J1810–197 (for example, due to a smaller initial wobble angle).

**Extended Data Fig. 5:**
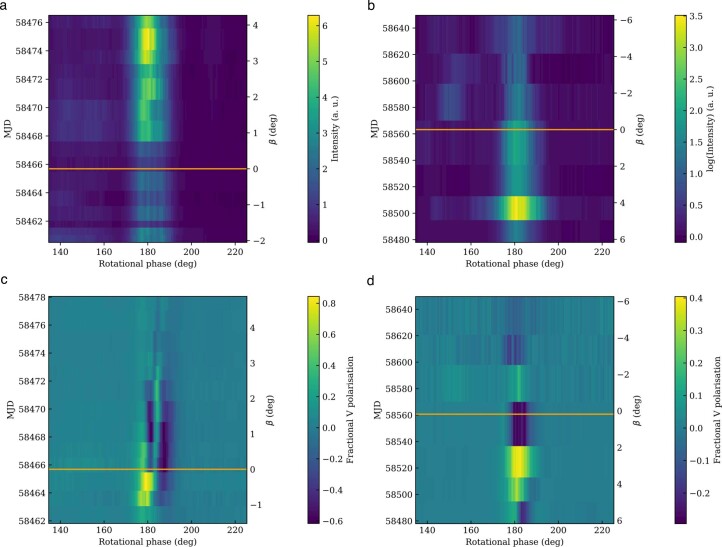
Temporal evolution of the polarisation of the pulse profile as our line of sight crosses the magnetic pole. The panels (a) and (b) show the total intensity as a function of time and rotational phase while panels (c) and (d) show the circular polarisation. Panels (a) and (c) show the pulse profile near the time of the first PA reversal, when *β* changes sign as shown by the orange line. Panels (b) and (d) show the pulse profile at the time of the second crossing of the magnetic pole. The *β*-scale on the y-axis is obtained from a linear fit to the *β* values shown in Fig. 2 in the limited MJD range of the plots.

The precession of XTE J1810–197 can potentially lead to changes in the observed polarimetric pulse profiles as our line of sight cuts through different parts of the beam. Additionally, free precession is expected to cause variations in the spin-down rate of XTE J1810–197, as the spin-down torque depends upon the angle *α*, varying approximately as ${\sin }^{2}\alpha$ (ref. ^47^). No large variations are observed in the shape of the pulse profile in the first week of observations, except for some intensity fluctuations, despite the large change in *β*, including a change of sign. However, while we only observe large amplitude variations in the left- and right-handed circular polarization mixed with propagation effects as the PA gradient first changes sign, we observe a reversal of the sign of the circular polarization at the time of the second crossing of the magnetic pole (Extended Data Fig. 5), as expected^48^ and observed in the case of the relativistic binary pulsar J1906+0746 (ref. ^18^). With our measured range of change of *α* (Fig. 2), we estimate the spin-down rate (Fig. 2 and equation 9 of ref. ^47^) to vary by about 700% to 30% for a plasma conductivity in the pulsar magnetosphere ranging from zero to infinity, respectively. However, we find no correlation between the spin-down rate presented in Fig. 3 of ref. ^35^ and *α*. This indicates that other physical processes such as the untwisting of magnetic field lines^2^, high-energy emission, the magnetar’s wind, magnetospheric flares or seismic activities^49^ must contribute more to variations in the spin-down rate of this magnetar than changes in the magnetic inclination angle.

The geometry of XTE J1810–197 has been independently constrained by the modelling of its X-ray pulse profile^50–52^. Assuming the X-ray spot axis is aligned with the magnetic axis (as valid for a dipolar magnetic field), we can compare the radio and X-ray derived geometry. We find our measurement of *α* is consistent with the results from the different analyses of X-ray data (if we take into consideration we use the historic convention of the PA increasing clockwise on the sky^53^ or if we refer to the opposite magnetic pole, that is *α* = 180° − *α*). The X-ray analyses, however, provide different and inconsistent estimates for *ζ* originating from the different assumptions behind their complex modelling. Our results rule out the modelling of the 2003 outburst by ref. ^50^ and strongly favour the analysis of ref. ^51^ based on, for example, thermal quiescent emission, a dipolar model for the magnetar surface temperature (instead of the uniform temperature assumed in ref. ^50^) and inclusion of light deflection from General Relativity. Combined with the Very Long Baseline Array estimate of the magnetar’s distance of $2.{5}_{-0.3}^{+0.4}$ kpc (ref. ^54^), our accurate measurement of *ζ* could eventually constrain the compactness (mass *M* over radius *R*) of the magnetar. Based on the modelling by ref. ^51^ assuming *M* = 1.4 *M*_⊙_ our constraint on *ζ* indicates *R* ≿ 15 km.

Neutron stars with asymmetric deformation along an axis different from the rotation axis are expected to emit gravitational waves (GW) at both once and twice their rotation frequency^55^. Numerous attempts have been made at detecting the GW emitted by normal and fast-spinning pulsars using ground-based interferometers such as the Laser Interferometer Gravitational-wave Observatory and Virgo. For slowly spinning NSs like magnetars and XTE J1810–197, the frequencies of the GW correspond to 0.18 and 0.36 Hz, falling in the frequency range (10^−4^ Hz to 1 Hz) of the Laser Interferometer Space Antenna gravitational wave observatory. However, the characteristic strain amplitude of the GW at the Earth is a function of ${P}_{{{{\rm{s}}}}}^{-2}$ (see, for example, ref. ^56^) and several orders of magnitude below the expected sensitivity of the Laser Interferometer Space Antenna gravitational wave observator.

Freely precessing magnetars were recently proposed^6,7^ to explain the periodicity observed in the activity of some repeating FRBs^57,58^. We have shown here that free precession of XTE J1810–197, probably excited at the time of its 2018 X-ray outburst, has been damped within a timescale of a few months, with a corresponding decay in both the wobble angle and the ellipticity. These results argue against free precession of magnetars as the mechanism behind repeating FRBs with activity periods of the order of months, at least for magnetars with *B*_surf_ similar to XTE J1810–197.

**Extended Data Fig. 6:**
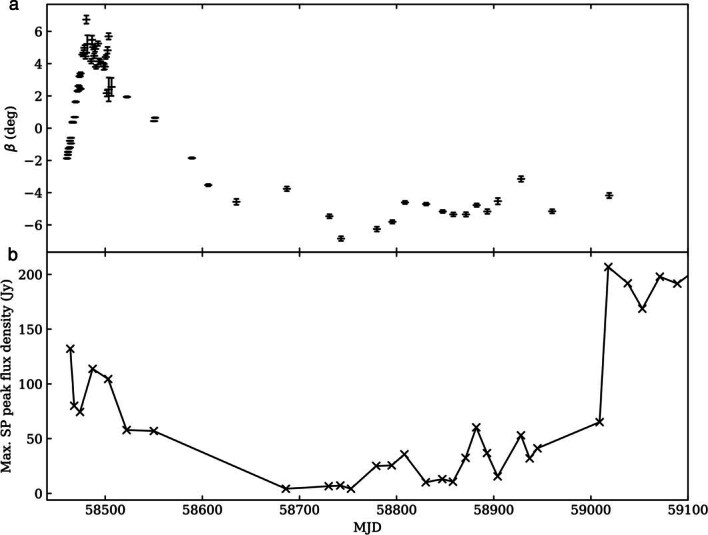
Temporal evolution of the maximum single pulse peak flux densities. In panel (a) we report the same *β* measurements as shown in Fig. 2. In panel (b), we show the maximum SP peak flux densities recorded at 6 GHz with black crosses.

Using our flux-calibrated single-pulse observations of XTE J1810–197 from Effelsberg, we investigated if the brightest single pulses (SPs) detected with peak flux density >200 Jy (still several orders of magnitude below the typical FRB luminosity^5^) were occurring at some specific impact parameter *β*. However, we find no link between *β* and the brightest SPs (Extended Data Fig. 6).

In summary, high-cadence radio and X-ray observations of magnetars, especially shortly after the detection of an outburst, are key to understanding the physics of free precession and could provide important information for testing the internal and magnetic structure of NSs.

## Methods

### Observations and data reduction

Following the detection of the radio revival of XTE J1810–197 on 8 December 2018, the Lovell Telescope at the Jodrell Bank Observatory (JBO) was used for a regular monitoring of the magnetar, at almost daily cadence during the first weeks of observations^11^. The JBO data were acquired with the ROACH pulsar backend^59^ tuned to a central frequency of 1,532 MHz (L-band). A 384 MHz bandwidth split over 768 channels was calibrated using a matrix template matching technique^60^ and observations of the pulsars B0540+23, B0611+22 and B1737–30. A total of 36 observations, folded with the ephemeris from ref. ^11^, were recorded between 8 December 2018 and 23 January 2019, before the telescope went down for maintenance. One additional observation with the Lovell Telescope was made on 9 March 2019, during an extended period of maintenance. Most JBO observations are between 30 to 60 min long.

At the Effelsberg observatory, we observed XTE J1810–197 with the S60 receiver of the 100 m telescope tuned to a frequency of 4.85 GHz (C-band) three days after the detection of its radio revival. Following this successful detection, we then started monitoring XTE J1810–197 with the S110 and S45 receivers tuned to frequencies of 2.55 GHz (S-band) and 6 GHz receivers (C/X-band), respectively, to provide complimentary frequency coverage to the JBO monitoring campaign. Since the S-band observations were less frequent and had a pulse profile with a lower signal-to-noise ratio (S/N) than the C/X-band observations, and were also recorded within 30 min of the C/X-band data, we decided to not include the S-band data in this study. Nonetheless we used the S-band data to verify the polarization calibration of the C/X-band data.

The circular-basis S110 and S60 receivers sent signals to the PSRIX backend^61^ and provided, respectively, 400 MHz and 500 MHz bandwidths split into 128 frequency channels. The PSRIX backend was configured to provide averaged pulse profiles folded over 10 s subintegrations across 1,024 phase bins using the rotational period determined from the most recently published timing ephemeris^11,35^. The data taken with the S45 linear-feed receiver were recorded with the PSRIX2 backend^62^ in PSRFITS search mode with 131 μs time resolution and 4 GHz of bandwidth centred at 6 GHz and split into 4,096 frequency channels. The data were folded offline across 2,048 phase bins using the latest ephemeris from ref. ^35^ to form a time-averaged pulse profile. The data were also folded across 16,384 phase bins to produce single-pulse archives. Twenty-five observations were recorded with the S45 receiver and the PSRIX2 backend, between 12 December 2018 and 18 June 2020.

All Effelsberg observations are usually between 10 to 20 min long. A 2 min scan of a polarized pulse noise diode recorded before each observation is used to calibrate the data in polarization with PSRCHIVE^63^. We routinely observed the planetary nebula NGC 7027 to flux-calibrate the Effelsberg observations.

All JBO and Effelsberg data were then corrected for Faraday rotation using the rotation measure of 74.44 rad m^−2^ value from ref. ^15^ and provide calibrated Stokes parameters *I*, *Q*, *U*, *V*^64^.

### Polarization of the pulse profiles

It has been previously reported^15^ that the PA of the linear polarization $L=\sqrt{{Q}^{2}+{U}^{2}}$ of XTE J1810–197 pulse profiles recorded since the 2018 outburst shows complex structure and deviates from the simple ʻS’-shaped curve predicted by the RVM^16^. The RVM is the standard geometrical model used by pulsar astronomers to interpret the PA and infer the viewing geometry of a pulsar, assuming a dipolar magnetic field.

Although the emission of the known radio magnetars has been shown to be usually highly linearly polarized, see, for example, refs. ^13,45,46^, we noticed some dips in the otherwise nearly 100% linearly polarized pulse profiles from multiple epochs at various phase ranges. This depolarization even occurs at different phase ranges for quasi-simultaneous observations at different frequencies (see, for example, the pulse profiles for MJD 58,474 in Supplementary Fig. 1). It is well known^65^ that the superposition of different polarization mode can lead to depolarization and bias in the PA of the average pulse profile. To mitigate this, we excluded the phase ranges where the fractional linear polarization of the pulse drops below 80%. A two-dimensional histogram of the observed PA values of the linear polarization of the single-pulse profiles (Supplementary Fig. 8) shows that the depolarization in *L* originates from the superposition of two different polarization modes and a PA branching, leading to some bias in the PA values after summation of the pulses. PA branching has been previously observed in the magnetar Swift J1818.0–1607 (ref. ^22^) and in a normal rotation-powered pulsar^66^. This frequency-dependent effect, attributed to birefringence in the star’s magnetosphere, was observed in the central part of the main component of the pulse starting around MJD 58,466 until it significantly decreased near MJD 58,500 (ref. ^67^). For the subsequent modelling of the PA data, these phase ranges were therefore excluded from the analysis.

### Comparing free and forced precession

The RVM, assuming the International Astronomical Union definition of the PA increasing counter-clockwise on the sky, can be written as:

1 $$\tan \left({\psi }_{0}-\psi \right)=\frac{\sin \alpha \sin \left(\phi -{\phi }_{0}\right)}{\sin \zeta \cos \alpha -\cos \zeta \sin \alpha \cos \left(\phi -{\phi }_{0}\right)},$$

with the viewing angle *ζ* = *α* + *β* (ref. ^17^). Here *α* is the magnetic inclination angle and *β* is the angle between the magnetic axis and our line of sight at the closest approach. The measured PA at any given pulse longitude *ϕ* is given by $\psi =\frac{1}{2}\arctan\left(\frac{U}{Q}\right)$. The parameter *ϕ*_0_ is the pulse longitude where the PA sweep is the steepest, that is, when *ψ* = *ψ*_0_ (the PA of the rotation axis projected on the plane of the sky).

In contrast to the data recorded in 2006–2008 where an interpulse preceded the main pulse by about 100° (refs. ^13,14^), RVM fits to the PA of the individual epochs recorded after 2018 can only poorly constrain *α* and *β*. Therefore, we performed simultaneous RVM fits to all epochs to model the temporal PA variations as either free or forced precession. For the forced precession scenario, we set a common *α* parameter across all epochs whereas for the free-precession scenario, *ζ* is the common parameter across all epochs. For each epoch, the three remaining RVM parameters *β*, *ϕ*_0_ and *ψ*_0_ are modelled independently.

We developed a set of codes based on modelRVM^68^ to simultaneously apply the RVM to the Stokes *Q* and *U* data of our dataset (instead of the traditional PA values) and explore the parameter space of the free and forced precession models with the nested sampling software PolyChord v.1.20.1 (ref. ^33^).

The analysis includes a scaling factor parameter per frequency band referred to as EFAC that acts as a multiplier factor to the Stokes *Q* and *U* off-pulse standard deviations. The dimensionality of the problem *N*_dim_ is therefore 1 + 3 × *N*_epochs _+ *N*_EFAC_, with *N*_epochs_ and *N*_EFAC_ being the number of epochs and the number of EFAC parameters included in the analysis, respectively. All RVM parameters are sampled using 10 × *N*_dim_ live points (with a minimum of 1,000 live points) from Gaussian priors with standard deviation of 5° (3° for *β*) and mean values derived from previous runs. We set the PolyChord *n*_repeats_ parameter (that is the slice sampling chain length used to produce a new live point) to 5 × *N*_dim_ to ensure we get reliable log-evidence ($\log {{{\mathcal{Z}}}}$) estimates from the nested sampling analysis. Each EFAC parameter is sampled from log-uniform prior in the ${\log }_{10}$ range [-0.4, 0.6]. The log-Bayes factor comes directly from the subtraction of $\log {{{\mathcal{Z}}}}$ between any two models. A log-Bayes factor >3 brings conclusive support for the model with the highest $\log$ ${{{\mathcal{Z}}}}$^69^.

In the following analyses, we selected the PA with phase bins that satisfy *L* > 3*σ*_*N*_, where *σ*_*N*_ is the off-pulse standard deviation, outside of the excluded phase ranges as described in the previous section.

We started by analysing the datasets from the two different radio bands independently (L and C/X bands) and then combined all the PA data from the two bands. The results are reported in Supplementary Table 1.

The log-Bayes factor are 54 for the JBO L-band and 9 for the Effelsberg C/X-band data in support of the free-precession interpretation of the PA. They thus provide very strong support the free-precession interpretation of the data. Unsurprisingly, the combined dataset also shows unarguable support for the free-precession scenario with a log-Bayes factor of 75.

As the free-precession model is the preferred model to explain the observed PA variations, we show in Supplementary Fig. 9 the posterior distributions for the parameter *ζ* from each analysis. The *ζ* posteriors from the two frequency bands and the combined dataset are consistent with each other. The posterior from the combined dataset gives a narrower distribution, with *ζ* = 161.0° ± 0.7°, arguing for XTE J1810–197 as being a near-aligned rotator. We show in Supplementary Fig. 10 the posterior distributions of the two EFAC parameters included in the analysis of the combined dataset. The mean values of the EFAC parameters are 1.12 and 1.44 for the L and C/X bands, respectively, indicating a good modelling of the PA.

### Twisted magnetic fields

It is assumed^2^ that the magnetic field of a magnetar can become twisted following some abrupt crustal motion of the star. The following slow and gradual untwisting of the magnetic field lines is presumed to power the observed outburst. The perturbation of the twisted field lines on the PA *ψ* can be written as^20^:

2 $${{\Delta }}{\psi }_{{{{\rm{twist}}}}}=-\frac{8}{9}\lambda {\sin }^{2}{\theta }_{{{{\rm{obs}}}}}$$

where *θ*_obs_ is the colatitude of the line of sight defined by

3 $$\cos {\theta }_{{{{\rm{obs}}}}}=\cos \alpha \cos \zeta +\sin \alpha \sin \zeta \cos \phi$$

and $\lambda =\pm \sqrt{\frac{35}{16}(1-n)}$. Here *n* ∈ [0, 1] defines the twist of the magnetic field lines. A positive *λ* indicates an eastward twist of the magnetic field lines in the star’s Southern hemisphere. Conversely, a negative *λ* corresponds to a westward twist. When *n* = 1, *λ* = 0 and the twist perturbation becomes null. As an example, we show in Supplementary Fig. 11 the predictions from the twisted RVM for a geometry representative of the results derived in the previous section.

Following the toy model by ref. ^70^, we describe the evolution of *n* during the outburst as an exponential decay of the form $n(t)=1-(1-{n}_{0}){\rm{e}}^{-t/{\tau }_{{{{\rm{t}}}}}}$ where *n*_0_ is the initial twist value at *t* = *T*_0_, the beginning of the 2018 outburst also assumed to be the start of the precession. *T*_0_ is constrained by the X-ray detection of the outburst^12^. The parameter *τ*_t_ represents the exponential decay timescale for the relaxation of the twisted magnetic field lines.

The results of the model comparison between free and forced precession including the twist perturbations (three additional parameters) are reported in Supplementary Table 2. They support with a log-Bayes factor of 322 the relaxation of an eastward twist of the magnetic field lines in the free-precession model as the preferred model. Extended Data Fig. 1 shows the waveform for the twist parameter *n*. These results suggest a very large twist of the magnetic field lines at the time of the outburst with $n=0.0{5}_{-0.05}^{+0.16}$ at a 99% confidence interval with *τ*_t_ > 730 days. The parameters *n*, *ζ* and *β* are covariant and with the twisted RVM, the value of the viewing angle has shifted to *ζ* = 167.3° ± 0.3°. The mean values of the EFAC parameters have decreased to 1.08 and 1.43 for the L and C/X bands, respectively.

### Emission height

The radio emission height *h*_em_ can be inferred from the offset Δ*ϕ*_A/R_ between the phase of the RVM inflection point (for example, *ϕ*_0_) and the centre of the pulse profile due to aberration and retardation effects^71^. It can be written as

4 $${h}_{{{{\rm{em}}}}}=\frac{{{\Delta }}{\phi }_{{{{\rm{A/R}}}}}}{4}{R}_{{{{\rm{LC}}}}}$$

where *R*_LC_ = *cP*_s_/2π is the light cylinder radius and *c* is the speed of light. The phases of the RVM inflection points are determined from the results of the free-precession modelling and the centre of the pulse is determined at the 4% and 0.5% total intensity levels for the JBO and Effelsberg data, respectively. We were able to derive emission heights for the Effelsberg data only from the first six epochs due to a drop of S/N for the subsequent epochs. Supplementary Fig. 12 shows the emission heights derived from the JBO and Effelsberg data. They show an increasing emission height in the range of 10,000 to 45,000 km with larger ∣*β*∣ values. Interestingly, these results are also consistent with the concept of a radius-to-frequency mapping^71,72^, with higher radio frequency emission emitted at lower altitudes. Most of the normal pulsar population have emission heights less than 1,000 km (ref. ^73^) but magnetars tend to have higher emission altitudes (Fig. 14 of ref. ^74^).

The radio emission altitude is therefore well above the neutron star surface. The ratio *h*_em_/*R* where *R* is the neutron star radius, typically of size 12 km, is larger than 1,000 in all observations. At these heights, the strength of the magnetic field significantly decreases from its value at the surface, at least by a factor ${({h}_{{{{\rm{em}}}}}/R)}^{3}$ for the dipole and a factor ${({h}_{{{{\rm{em}}}}}/R)}^{\ell +2}$ for a multipole of order *ℓ* ≥ 2, the dipole being a *ℓ* = 1 multipole. The surface dipole magnetic field strength is estimated to be around *B*_surf_ ≈ 2.7 × 10^10^ T (ref. ^44^). At the radio emission site it is a billion times lower, amounting to a few Tesla or less. Even if quadrupolar surface magnetic fields are dominant at the surface, remaining dominant at an altitude of thousands of stellar radii would require a strength at the surface of at least 1,000 times larger than the dipole. Therefore, a physical process should maintain such a strong multipolar field for a time at least equal to the age of the magnetar. This is a stringent constraint on the magnetic field evolution and decay, difficult to explain with our current understanding of neutron star magnetic fields. Ref. ^75^ extensively discusses on the impact of multipolar fields on the neutron star spin-down luminosity. It has recently been shown^76^ that adding a multipolar magnetic field component to the dipole does not alter the RVM PA expectations. However their results rely on an axisymmetric configuration where the multipole is oriented with respect to the dipole in such a way that the planes containing the field lines remain unchanged. If the orientation of this multipole would be random compared to the dipole, which is a more realistic configuration, and if this multipole would remain dominant at the radio emission heights derived above (a highly unlikely case) the RVM PA would be significantly altered. As a consequence, the impact of a multipole component can be confidently ignored when studying the PA evolution in radio.

### Correlation between spin-down and *α*

The spin-down rate of neutron stars (hereafter referred as $\dot{\nu }$ similarly to the pulsar timing literature) is thought to vary approximately with ${\sin }^{2}\alpha$ (for example, ref. ^47^). We investigate here this correlation. We decided on using the Spearman correlation coefficient due to the non-Gaussian distribution of the data. To apply it, we first estimated $\dot{\nu }$ at the epochs of our dataset by linearly interpolating the $\dot{\nu }$ measurements from ref. ^35^ before computing the Spearman correlation coefficient *ρ*. The *α* measurements outside of the $\dot{\nu }$ measurements window were discarded. We find *ρ* = −0.03 with a *P*value of 0.86, where the null hypothesis is that our two datasets are linearly uncorrelated. This result, shown in Supplementary Fig. 13 supports the interpretation that we see no apparent correlation between the spin-down and the variation of geometry or that this variation is not contributing significantly to the spin-down compared to the intrinsic rotational instability commonly observed in magnetars.

### Reprocessing of the Effelsberg 2006 archival data

XTE J1810–197 was observed in the summer of 2006 with Effelsberg as part of a multifrequency observing campaign with the Lovell and Westerbork telescopes to study the magnetar’s polarization^13^. In this work, the PA data from the two main pulse components observed in 2006 was fitted independently to the RVM, but assuming a common *ζ*. For the observation recorded on MJD 53,934 at 8.4 GHz (session 3), a RVM fit gave *ζ* = 83° using the historical RVM convention^53^. This result would translate into *ζ* = 180 − *ζ*_*o*_ = 97° when referring to the same IAU definition of the PA as used in this work. The 2006 result is therefore in stark contrast to our current value of *ζ*. We therefore decided to revisit the 2006 RVM results. We chose to focus on the Effelsberg 8.4 GHz observations as they exhibit the profiles with the highest S/N^13^. We selected the four epochs recorded at 8.4 GHz with the highest S/N where both components were observed, that is MJDs 53,926, 53,934, 53,938 and 53,944.

We performed separate RVM fits to these four epochs using MODELRVM, including in each case the four free parameters of the RVM plus an EFAC parameter. We used all PA data points with *L* > 4*σ*_*N*_, except for MJD 53,944 where high level of linear depolarization is observed in the second component (see also Fig. 9 of ref. ^13^) and the corresponding phase range was excluded from the fit. Extended Data Fig. 2 shows the four selected polarimetric pulse profiles used to fit the PA data and Extended Data Fig. 3 shows the RVM posterior distributions for each epoch. Supplementary Table 3 summarizes the results of the modelling. We find that all modelled epochs from 2006 provide results on *ζ* consistent with our new dataset. We also find the angle *β* to be constant between all four epochs within our error bars. We interpret the discrepancy between these new results and the results published in ref. ^13^ as being due to the exclusion of the PA data when depolarization is observed in the second pulse component.

We also applied the twisted RVM to each of the four epochs to try to measure the twist parameter for these observations taken two years before the radio disappearance of the magnetar. Unfortunately, the model could not constrain the twist parameter for any of the epochs and we attribute this to the narrowness of the pulse profile components in 2006.

Data from the Parkes radio telescope recorded in 2006 were also used to derive the geometry of XTE J1810–197 (ref. ^14^). Correcting for the likely use of the previous older convention for the RVM^53^, their results (no uncertainties were provided) would translate to *α* ≈ 176° and *β* ≈ −4°, in broad agreement with our reprocessing of the Effelsberg 2006 data.

### Theoretical models

#### Free precession of neutron stars

To set the body into free precession, a rotating NS must have some deformations misaligned with the centrifugal bulge. We can write the moment of inertia tensor of the NS as a sum of a spherical and two quadrupolar parts^77,78^

5 $${{{I}}}={I}_{0}{{{\delta }}}+{{\Delta }}{I}_{{{{\upomega }}}}\left({{{{{\mathbf{n}}}}}}_{{{{\upomega }}}}{{{{{\mathbf{n}}}}}}_{{{{\upomega }}}}-{{{\delta }}}/3\right)+{{\Delta }}{I}_{{{{\rm{d}}}}}\left({{{{{\mathbf{n}}}}}}_{{{{\rm{d}}}}}{{{{{\mathbf{n}}}}}}_{{{{\rm{d}}}}}-{{{\delta }}}/3\right)\,.$$

The first term on the right-hand side is the spherical part of the non-rotating undeformed star with *δ* being the unit tensor. The second term is the change due to centrifugal forces, and has the unit vector **n**_ω_ as the symmetric axis along the star’s angular velocity **ω**. The third term is the change due to crustal shear stresses or magnetic fields, which is assumed to be axisymmetric along a unit vector **n**_d_ for simplicity.

The angular momentum is then related to the angular velocity via

6 $${{{{\mathbf{L}}}}}=\left({I}_{0}+2/3{{\Delta }}{I}_{{{{{{\upomega }}}}}}-1/3{{\Delta }}{I}_{{{{\rm{d}}}}}\right){{{{\mathbf{\upomega}}}}}+{{\Delta }}{I}_{{{{\rm{d}}}}}{{\mathbf{\upomega }}}_{3}{{{{{{\mathbf{n}}}}}}}_{{{{\rm{d}}}}}\,.$$

This shows that **L**, **ω**, and **n**_d_ are coplanar. As the angular momentum is conserved in free precession, this plane must rotate around **L**. Taking the components of **L**, we obtain

7 $${L}_{1}=\left({I}_{0}+2/3{{\Delta }}{I}_{{{{\upomega }}}}-1/3{{\Delta }}{I}_{{{{\rm{d}}}}}\right){\omega }_{1}\equiv {I}_{1}{\omega }_{1}\,,$$

8 $${L}_{2}=\left({I}_{0}+2/3{{\Delta }}{I}_{{{{\upomega }}}}-1/3{{\Delta }}{I}_{{{{\rm{d}}}}}\right){\omega }_{2}\equiv {I}_{1}{\omega }_{2}\,,$$

9 $${L}_{3}=\left({I}_{0}+2/3{{\Delta }}{I}_{{{{{{\upomega }}}}}}+2/3{{\Delta }}{I}_{{{{\rm{d}}}}}\right){\omega }_{3}\equiv {I}_{3}{\omega }_{3}\,.$$

Despite the triaxiality of *I*, the components of the angular momentum are formally equivalent to a rigid symmetric rotator with *I*_3_ − *I*_1_ = Δ*I*_d_. We define the ellipticity of the deformed NS as

10 $$\epsilon \equiv \frac{{I}_{3}-{I}_{1}}{{I}_{1}}=\frac{{{\Delta }}{I}_{{\mathrm{d}}}}{{I}_{1}}\,.$$

In the body frame, the equation of motion of a freely precessing body can be described by the Euler equation

11 $${\dot{\mathbf{L}}}+{{{\mathbf{\upomega} }}}\times {{{{\mathbf{L}}}}}=0\,,$$

where the over dot denotes the derivative with respect to the time *t*. The equation of motion only involves **L** and **ω**. Thus, equations (7)–(9) indicate that the precession of the triaxial body is formally equivalent to that of a rigid symmetric top^78^. According to equation (11), the components of the angular velocities satisfy

12 $${\dot{\omega }}_{1}=-\epsilon {\omega }_{2}{\omega }_{3}\,,$$

13 $${\dot{\omega }}_{2}=\epsilon {\omega }_{1}{\omega }_{3}\,,$$

14 $${\dot{\omega }}_{3}=0.$$

The solutions of equations (12)–(14) are

15 $${\omega }_{1}=a\cos ({\omega }_{{{{\rm{p}}}}}t+{\beta }_{0})\,,$$

16 $${\omega }_{2}=a\sin ({\omega }_{{{{\rm{p}}}}}t+{\beta }_{0})\,,$$

17 $${\omega }_{3}=b\,,$$

where *a* and *b* are constants satisfying *a*^2^ + *b*^2^ = *ω*^2^, *ω*_p_ = *ϵω*_3_ is the angular frequency and *β*_0_ is an initial phase.

We introduce standard Euler angles to describe the orientation of the NS in the inertial frame, with the polar axis along the angular momentum **L**. Let *η* and *θ* denote the azimuthal and polar angles of the deformation axis **n**_d_, and *λ* represent a rotation about **n**_d_. As *ω*_3_ and **L** are constant, the wobble angle *θ* is also constant during the precession. We label the angle between **ω** and **L** as *θ*_1_, which satisfies

18 $$\tan (\theta +{\theta }_{1})=\frac{{I}_{3}}{{I}_{1}}\tan \theta =(1+\epsilon )\tan \theta \,.$$

Expanding the above equation to the first order of *ϵ*, we obtain

19 $${\theta }_{1}=\epsilon \sin \theta \cos \theta \,.$$

This angle is much smaller than *θ* because *ϵ* is extremely small. Therefore, we can neglect *θ*_1_ when evaluating the geometry of the star. To get the motion in the inertial frame, we decompose the angular velocity into

20 $${{{\mathbf{\upomega} }}}=\dot{\eta }{{{{{\mathbf{n}}}}}}_{{{{\rm{L}}}}}+\dot{\lambda }{{{{{\mathbf{n}}}}}}_{{{{\rm{d}}}}}\,,$$

where **n**_L_ is the unit vector along **L** and $\dot{\eta }$ and $\dot{\lambda }$ are the time derivatives of *η* and *λ*, respectively. Substituting this equation into equation (6), we get

21 $${{{\bf{L}}}}={I}_{1}\dot{\eta }\,,$$

22 $$\dot{\lambda }=-\epsilon {\omega }_{3}=-{\omega }_{{{{\rm{p}}}}}\,.$$

Thus, as viewed from the inertia frame, the deformation axis rotates at a rate of $\dot{\eta }$ about **L** in a cone of half-angle *θ*. We refer this angular frequency as the inertial precession frequency. Superimposed upon $\dot{\eta }{{{{{{\bf{n}}}}}}}_{{{{\rm{L}}}}}$ is a rotation about the deformation axis at a rate of $\dot{\lambda }=-{\omega }_{{{{\rm{p}}}}}$. We refer to *ω*_p_ as the free-precession frequency and define the free-precession period as

23 $${P}_{{{{\rm{f}}}}}=\frac{2\uppi }{| {\omega }_{{{{\rm{p}}}}}| }\simeq \frac{{P}_{{{{\rm{s}}}}}}{| \epsilon \cos \theta | }\,,$$

where *P*_s_ = 2π/*ω* is the spin period.

The magnetic inclination *α* between **L** and the magnetic dipole **m** can be represented as

24 $$\cos \alpha =\sin \theta \sin \lambda \sin \chi +\cos \theta \cos \chi \,,$$

where *χ* is the angle between **m** and **n**_d_, and the precession phase *λ* is

25 $$\lambda =\arctan \frac{{\omega }_{1}}{{\omega }_{2}}=\frac{\uppi }{2}-{\omega }_{{{{\rm{p}}}}}t-{\beta }_{0}\,.$$

The precession phase *λ* changes with time as long as *χ* ≠ 0, which leads to the periodic variations of *α* during free precession. However, for XTE J1810–197, the variations of *α* damp away in less than a precession period. The simple free precession cannot explain this phenomenon. In the following, we investigate damped-precession models based on the free-precession solution.

#### Free precession with relaxing ellipticity model

In this model, we expand on the geometry of a freely precessing NS to consider a decreasing ellipticity *ϵ* of the NS such that *P*_f_ becomes increasingly longer. The star gradually relaxes to a spherical shape. As a result, the precession phase is frozen at a certain epoch and *α* turns into constant. We assume

26 $${I}_{3}={I}_{1}[1+\epsilon (t)]\,,$$

with $\dot{\epsilon } < 0$. In this case, the Euler equations are

27 $${\dot{\omega }}_{1}=-\epsilon {\omega }_{2}{\omega }_{3}\,,$$

28 $${\dot{\omega }}_{2}=\epsilon {\omega }_{1}{\omega }_{3}\,,$$

29 $${\dot{\omega }}_{3}=-\frac{\dot{\epsilon }\,{\omega }_{3}}{1+\epsilon }\,.$$

We can neglect the change of *ω*_3_ and *θ* when studying the geometry because the ellipticity is very small. In this approximation, the components of **ω** can be expressed as

30 $${\omega }_{1}(t)=a\cos \left[g(t)+{\beta }_{0}\right]\,,$$

31 $${\omega }_{2}(t)=a\sin \left[g(t)+{\beta }_{0}\right]\,,$$

32 $${\omega }_{3}(t)=b\,,$$

where

33 $$g(t)=b\int\nolimits_{0}^{t}\epsilon (t){{{\rm{d}}}}t\,.$$

The angle *λ* is

34 $$\lambda =\arctan \frac{{\omega }_{1}}{{\omega }_{2}}=\frac{\uppi }{2}-g(t)-{\beta }_{0}\,.$$

For simplicity, we assume that the ellipticity changes in an exponential form

35 $$\epsilon (t)={\epsilon }_{0}{\rm{e}}^{-t/{\tau }_{\epsilon }}\,,$$

where *τ*_ϵ_ is the relaxation timescale of the ellipticity. In this parametrization, the function *g*(*t*) is

36 $$g(t)=b{\epsilon }_{0}\tau -b{\rm{e}}^{-\frac{t}{{\tau }_{\epsilon }}}{\epsilon }_{0}\tau \,.$$

#### Phenomenological model of decaying wobble angle

The decay of the wobble angle can also make the precession damp away, which is closely related to the internal couplings between the core and the crust. For simplicity, we first take a phenomenological model in which the wobble angle decays in an exponential form

37 $$\theta (t)={\theta }_{0}{\rm{e}}^{-t/{\tau }_{{{{\rm{\theta }}}}}}\,,$$

and the precession phase evolves in the free-precession form

38 $$\dot{\lambda }(t)=-\epsilon \omega \cos \theta (t)\,,$$

where *τ*_θ_ is the decaying timescale of the wobble angle.

#### Phenomenological model of decaying wobble angle and relaxing ellipticity

The phenomenological model of decaying wobble angle is unable to fit the data well. Therefore, we combined the decay of the wobble angle with the relaxing ellipticity. We considered two cases for the ellipticity decrease to represent a possible constant contribution to *ϵ*,

39 $$\epsilon (t)={\upepsilon }_{1}{\rm{e}}^{-t/{\tau }_{{{\upepsilon }}}}\,,$$

and

40 $$\epsilon (t)={\upepsilon }_{0}+{\upepsilon }_{1}{\rm{e}}^{-t/{\tau }_{{{\upepsilon }}}}\,,$$

where *ϵ*_0_ and *ϵ*_1_ are constant, and *τ*_ϵ_ is the relaxing timescale of the ellipticity. In this model, the precession phase is

41 $$\lambda (t)=\frac{\uppi }{2}-\int\nolimits_{0}^{t}\epsilon (t)\omega \cos \theta (t)\,{{{\rm{d}}}}t-{\beta }_{0}\,.$$

#### Model of crust–core frictional coupling combined with relaxing ellipticity

In this model, we replace the phenomenological description of the decay of the wobble angle with a frictional coupling between the core and the crust of the NS. Following refs. ^79–81^, we take a two-component model for the NS. The component which is coupled directly with the external torque **N**_ext_ consists of the crust and the charged fluids in the core. The other component, which contains most of the moment of inertia, is the fluid in the core of the star. We do not consider any superfluid-pinning to the crust, because pinning will inevitably make the precession very fast^25,80^, which is contradictory to our observations. The two components are coupled through an internal torque **N**_int_. We label the two components with ʻcʼ for the crust and ʻfʼ for the fluid in the core.

Standing in the corotating frame of the crust, the Euler equations describing the motion of the angular frequency vector **ω**_c_ and **Ω**_f_ can be written as

42 $${\dot{\mathbf{L}}}_{\rm{c}}+{\mathbf{\upomega}}_{\rm{c}}\times {\mathbf{L}}_{\rm{c}}={\mathbf{N}}_{\rm{ext}}+{\mathbf{N}}_{\rm{int}}\,,$$

43 $${\dot{\mathbf{L}}}_{{{{\rm{f}}}}}+{{{{\mathbf{\Omega }}}_{{{{\rm{f}}}}}}}\times {{{{{\mathbf{L}}}}}}_{{{{\rm{f}}}}}=-{{{{{\mathbf{N}}}}}}_{{{{\rm{int}}}}}\,,$$

where **L**_c_ and **L**_f_ are the angular momentum of the two components. We ignore **N**_ext_ since it only has large effects on the orientation of the star in spin-down timescale (it does play an important role on the spin evolution).

In general, the precession of the fluid core is driven by an internal torque depending on the rotational velocity lags between the solid and the fluid. With this velocity dependent torque, the angular momentum of the system is conserved but the kinetic energy is dissipated. The precession of the crust will be damped with decaying wobble angle. We take the internal torque as

44 $${{{{{\mathbf{N}}}}}}_{{{{\rm{int}}}}}=K({{{{\mathbf{\Omega }}}}}_{{{{\rm{f}}}}}-{{{{{\mathbf{\upomega }}}}}}_{{{{\rm{c}}}}})\,,$$

where *K* is a constant depending on the microscopic physics of the frictional coupling. Then the Euler equations for the components of **ω**_c_ are

45 $${\dot{\omega }}_{1}=-\epsilon {\omega }_{2}{\omega }_{3}-\frac{1}{{\tau }_{c}}\left({\omega }_{1}-{{{\Omega }}}_{1}\right)\,,$$

46 $${\dot{\omega }}_{2}=\epsilon {\omega }_{1}{\omega }_{3}-\frac{1}{{\tau }_{c}}\left({\omega }_{2}-{{{\Omega }}}_{2}\right)\,,$$

47 $${\dot{\omega }}_{3}=-\frac{1}{(1+\epsilon ){\tau }_{c}}\left({\omega }_{3}-{{{\Omega }}}_{3}\right)-\frac{{u}_{3}\dot{\epsilon }}{1+\epsilon }\,,$$

where *ω*_1_, *ω*_2_ and *ω*_3_ are the components of **ω**_c_, Ω_1_, Ω_2_ and Ω_3_ are the components of **Ω**_f_, and *τ*_c_ = *I*_c_/*K* is the timescale for the frictional coupling. The Euler equations for the components of **Ω**_f_ are

48 $${\dot{\Omega }}_{1}=-\left({\omega }_{2}{{{\Omega }}}_{3}-{\omega }_{3}{{{\Omega }}}_{2}\right)+\frac{\kappa }{{\tau }_{c}}\left({\omega }_{1}-{{{\Omega }}}_{1}\right)\,,$$

49 $${\dot{\Omega }}_{2}=-\left({\omega }_{3}{{{\Omega }}}_{1}-{\omega }_{1}{{{\Omega }}}_{3}\right)+\frac{\kappa }{{\tau }_{c}}\left({\omega }_{2}-{{{\Omega }}}_{2}\right)\,,$$

50 $${\dot{\Omega }}_{3}=-\left({\omega }_{1}{{{\Omega }}}_{2}-{\omega }_{2}{{{\Omega }}}_{1}\right)+\frac{\kappa }{{\tau }_{c}}\left({\omega }_{3}-{{{\Omega }}}_{3}\right)\,,$$

where *κ* = *I*_c_/*I*_f_ is the ratio between the moment of inertia of the crust and the core.

In principle, one can integrate equations (45)–(50) with appropriate initial conditions. However, it is very slow to integrate those six equations directly. To do parameter estimation more efficiently, we first make the following transformations

51 $$\left(\begin{array}{l}{u}_{1}\\ {u}_{2}\\ {u}_{3}\end{array}\right)=\left(\begin{array}{ccc}\cos \gamma &-\sin \gamma &0\\ \sin \gamma &\cos \gamma &0\\ 0&0&1\end{array}\right)\left(\begin{array}{l}{\omega }_{1}\\ {\omega }_{2}\\ {\omega }_{3}\end{array}\right)\,,$$

and

52 $$\left(\begin{array}{l}{v}_{1}\\ {v}_{2}\\ {v}_{3}\end{array}\right)=\left(\begin{array}{ccc}\cos \gamma &-\sin \gamma &0\\ \sin \gamma &\cos \gamma &0\\ 0&0&1\end{array}\right)\left(\begin{array}{c}{{{\Omega }}}_{1}\\ {{{\Omega }}}_{2}\\ {{{\Omega }}}_{3}\end{array}\right)\,.$$

Here the angle *γ* is defined as

53 $$\gamma (t)=\int-\epsilon {\omega }_{3}{{{\rm{d}}}}t\,.$$

Then the Euler equations can be written as

54 $${\dot{u}}_{1}=-\frac{1}{{\tau }_{{\mathrm{c}}}}\left({u}_{1}-{v}_{1}\right)\,,$$

55 $${\dot{u}}_{2}=-\frac{1}{{\tau }_{{\mathrm{c}}}}\left({u}_{2}-{v}_{2}\right)\,,$$

56 $${\dot{u}}_{3}=-\frac{1}{{\tau }_{{\mathrm{c}}}(1+\epsilon )}\left({u}_{3}-{v}_{3}\right)-\frac{{u}_{3}\dot{\epsilon }}{1+\epsilon }\,,$$

57 $${\dot{v}}_{1}=\frac{\kappa }{{\tau }_{{\mathrm{c}}}}\left({u}_{1}-{v}_{1}\right)-\left({u}_{2}{v}_{3}-{u}_{3}{v}_{2}\right)+\epsilon {u}_{3}{v}_{2}\,,$$

58 $${\dot{v}}_{2}=\frac{\kappa }{{\tau }_{{\mathrm{c}}}}\left({u}_{2}-{v}_{2}\right)-\left({u}_{3}{v}_{1}-{u}_{1}{v}_{3}\right)-\epsilon {u}_{3}{v}_{1}\,,$$

59 $${\dot{v}}_{3}=\frac{\kappa }{{\tau }_{{\mathrm{c}}}}\left({u}_{3}-{v}_{3}\right)-\left({u}_{1}{v}_{2}-{u}_{2}{v}_{1}\right)\,.$$

Further, we take

60 $$\epsilon {{{\delta }}}={{{u}}}-{{{v}}}\,.$$

For XTE J1810–197, the damping timescale of the precession *τ*_d_ is on the order of *P*_f_. According to the Bondi–Gold relation^79^,

61 $$\frac{{P}_{{{{\rm{s}}}}}}{{P}_{{{{\rm{f}}}}}} \approx \frac{{\tau }_{{\mathrm{c}}}}{{\tau }_{{{{\rm{d}}}}}}\,,$$

we quickly notice that *τ*_c_ ≈ *P*_s_. The crust and the fluid core are so strongly coupled that *u* and *v* become nearly aligned in the timescale of *τ*_c_, with a difference on the order of *ϵ*. Besides, the relaxing timescale of the ellipticity *τ*_ϵ_ ≫ *τ*_c_. Thus, we can neglect the higher order contributions and set ${\dot{\delta}}=0$. In this approximation, the Euler equations are

62 $${\dot{u}}_{1}=-\frac{\epsilon }{{\tau }_{{\mathrm{c}}}}{\delta }_{1}\,,$$

63 $${\dot{u}}_{2}=-\frac{\epsilon }{{\tau }_{{\mathrm{c}}}}{\delta }_{2}\,,$$

64 $${\dot{u}}_{3}=-\frac{\epsilon }{{\tau }_{{\mathrm{c}}}}{\delta }_{3}\,,$$

65 $$0=-\frac{1+\kappa }{{\tau }_{{\mathrm{c}}}}{\delta }_{1}+{u}_{3}{\delta }_{2}-{u}_{2}{\delta }_{3}-{u}_{2}{u}_{3}\,,$$

66 $$0=-{u}_{3}{\delta }_{1}-\frac{1+\kappa }{{\tau }_{{\mathrm{c}}}}{\delta }_{2}+{u}_{1}{\delta }_{3}+{u}_{3}{u}_{1}\,,$$

67 $$0={u}_{2}{\delta }_{1}-{u}_{1}{\delta }_{2}-\frac{1+\kappa }{{\tau }_{{\mathrm{c}}}}{\delta }_{3}\,.$$

We have three differential equations and three algebraic equations, which can be solved very fast. Once we integrate out *u* and *δ*, the angular frequencies **ω**_c_ and **Ω**_f_ can be obtained by a rotation transformation. Then the precession phase can be obtained from the relation $\lambda =\arctan \frac{{\omega }_{1}}{{\omega }_{2}}$. We considered again the same description for the decay of *ϵ* as in model 3, with equations (39) and (40).

### Running the free-precession models

To perform the comparison between the different free-precession models described above, we again apply the RVM to the same Stokes *Q* and *U* data as used in the comparison between free and forced precession. Across all epochs, *ζ* remains the common RVM parameter. All priors for the parameters in the free-precession models are described in Supplementary Table 4. In all models, we assume *P*_s_ to be constant after checking that taking into account $\dot{{P}_{{{{\rm{s}}}}}}$ does not affect the results. For each model, we also compared the Bayesian log-evidence $\log {{{\mathcal{Z}}}}$ with and without including the toy model for a twisted magnetosphere described previously. We report $\log {{{\mathcal{Z}}}}$ from all models in Extended Data Table 1. The preferred model with a Bayes factor of 45 is the phenomenological model of wobble angle decay with relaxing ellipticity to non-zero value. The posteriors and covariances between the parameters are shown in Supplementary Fig. 14. The next preferred model is the model based on frictional crust–core coupling combined with relaxing ellipticity to a non-zero value. The posteriors and covariances between the parameters of this model are shown in Supplementary Fig. 15.

### Supplementary information

Supplementary Information Supplementary Figs. 1–15 and Tables 1–4.

## Figures and Tables

**Extended Data Table 1:**
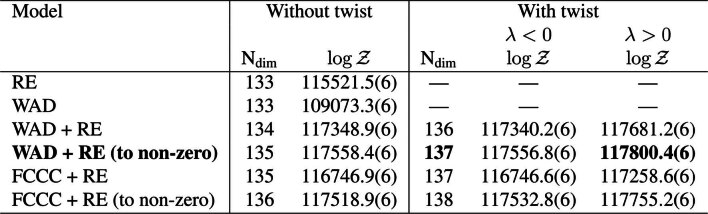
Comparison between the different models of free precession, including the effects of twisted magnetic fields

The acronyms RE, WAD and FCCC refer to the models of relaxing ellipticity, phenomenological wobble angle decay and frictional crust-core coupling. The two least likely models (RE and WAD) were not run with the twisted RVM hence the missing values.

## Data Availability

The polarimetric profiles can be downloaded via the Max Planck Digital Library: https://keeper.mpdl.mpg.de/d/1ab5be1fcf974027bf07/.

## Code availability

The modelling codes can be downloaded from https://github.com/gdesvignes/XTE_J1810-197.
