# Supplementary material for: A freely precessing magnetar following an X-ray outburst
Source: Nat Astron. 2024 Apr 8;8(5):617–27. doi: 10.1038/s41550-024-02226-7 (PMC11111412; doi:10.1038/s41550-024-02226-7)
Supplement: Supplementary file 1 — Supplementary Figs. 1–15 and Tables 1–4. [file 41550_2024_2226_MOESM1_ESM.pdf]

nature astronomy

Article

https://doi.org/10.1038/s41550-024-02226-7

A freely precessing magnetar following an X-ray outburst

In the format provided by the authors and unedited

Supplementary Information

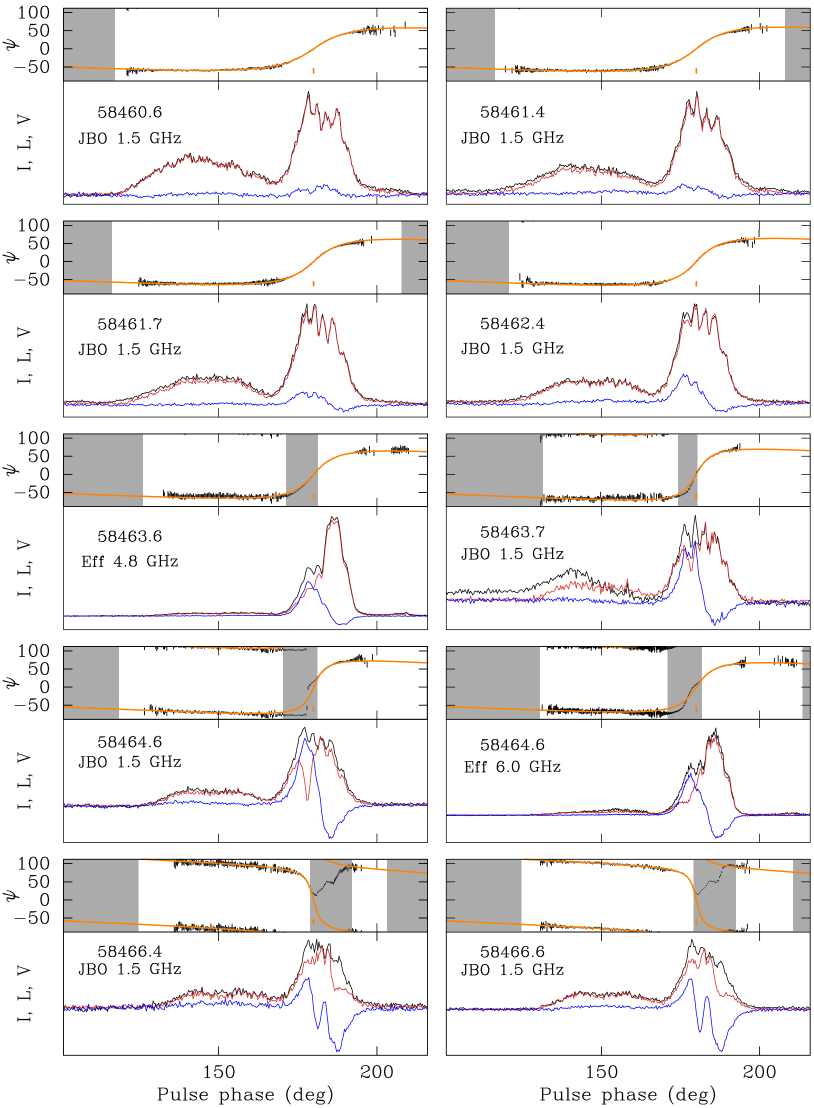

Supplementary Figure 1: Polarised pulse profiles of XTE J1810−197 recorded between MJDs 58460.6 and 58466.6. Top panel: the black error bars represent the PA data while the grey background show the phase ranges where PA data have been excluded from the fit. The uncertainty in the PA is calculated given L and the off-pulse standard deviation of I [1]. The oranges curves show the RVM prediction from the maximum likelihood parameters. The orange horizontal error bar at phase 180° indicates the 95% confidence level on the one-dimensional marginalised posterior of ϕ_0, the position of the RVM inflection point. Bottom panel: The black, red and blue lines show the total intensity, linear and circular polarisation, respectively. The MJD is indicated on the left of the pulse with the telescope and central observing frequency. The pulse profiles are aligned such that the inflection point of the RVM (i.e. $\phi_0 = 0°$) from the free precession analysis is set at the pulse phase (x-axis of the figure) of 180°.

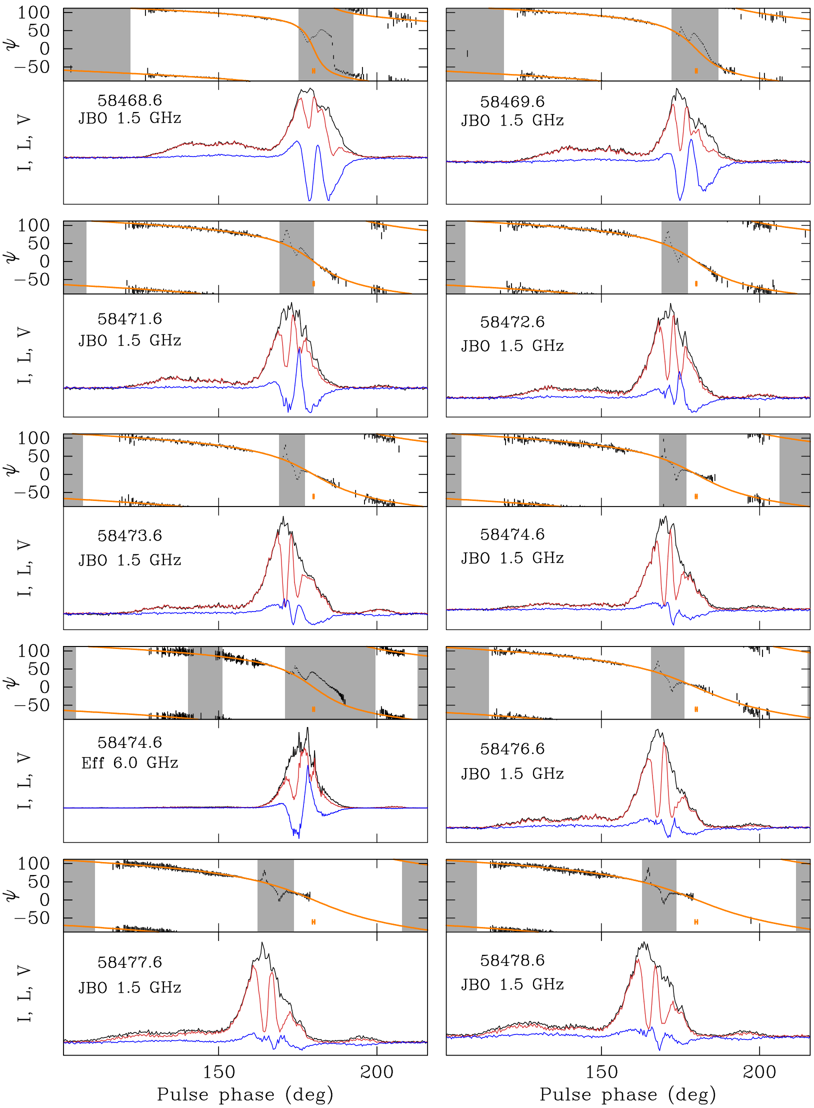

Supplementary Figure 2: Polarised pulse profiles of XTE J1810−197 recorded between MJDs 58468.6 and 58478.6. Top panel: the black error bars represent the PA data while the grey background show the phase ranges where PA data have been excluded from the fit. The uncertainty in the PA is calculated given L and the off-pulse standard deviation of I [1]. The oranges curves show the RVM prediction from the maximum likelihood parameters. The orange horizontal error bar at phase 180° indicates the 95% confidence level on the one-dimensional marginalised posterior of ϕ_0, the position of the RVM inflection point. Bottom panel: The black, red and blue lines show the total intensity, linear and circular polarisation, respectively. The MJD is indicated on the left of the pulse with the telescope and central observing frequency. The pulse profiles are aligned such that the inflection point of the RVM (i.e. $\phi_0 = 0°$) from the free precession analysis is set at the pulse phase (x-axis of the figure) of 180°.

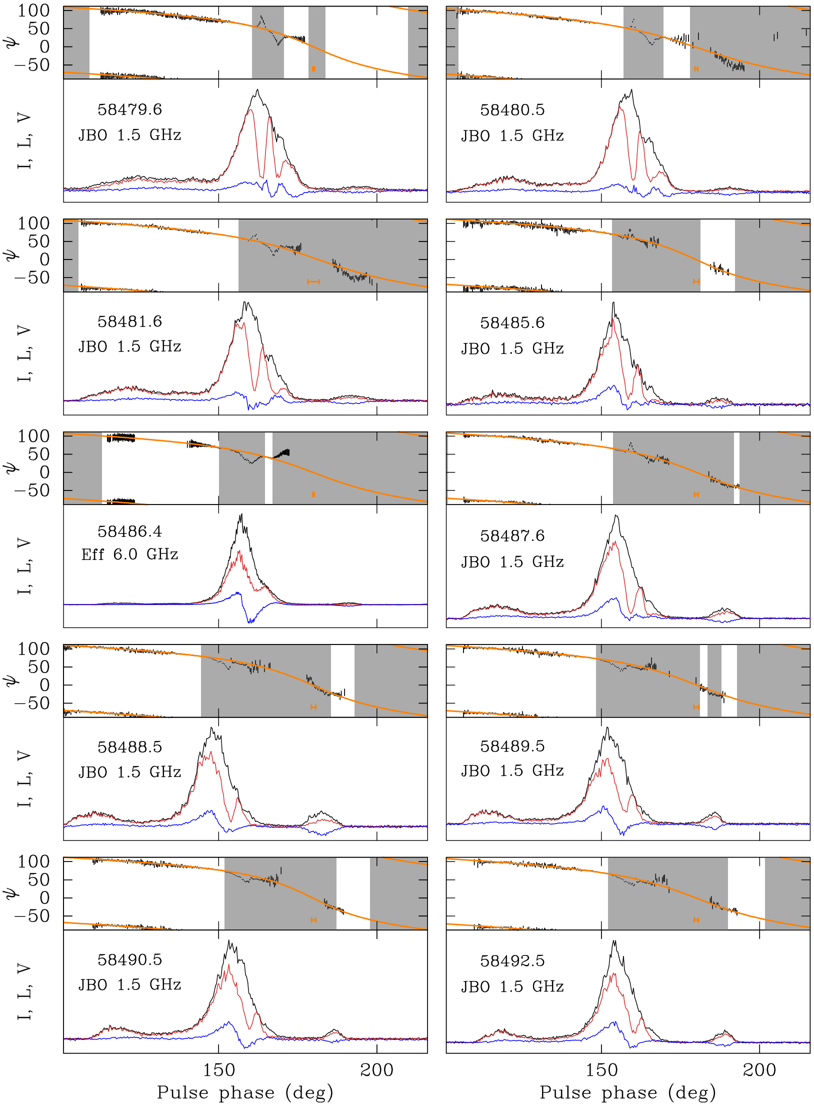

Supplementary Figure 3: Polarised pulse profiles of XTE J1810−197 recorded between MJDs 58479.6 and 58492.5. Top panel: the black error bars represent the PA data while the grey background show the phase ranges where PA data have been excluded from the fit. The uncertainty in the PA is calculated given L and the off-pulse standard deviation of I [1]. The oranges curves show the RVM prediction from the maximum likelihood parameters. The orange horizontal error bar at phase 180° indicates the 95% confidence level on the one-dimensional marginalised posterior of ϕ_0, the position of the RVM inflection point. Bottom panel: The black, red and blue lines show the total intensity, linear and circular polarisation, respectively. The MJD is indicated on the left of the pulse with the telescope and central observing frequency. The pulse profiles are aligned such that the inflection point of the RVM (i.e. $\phi_0 = 0°$) from the free precession analysis is set at the pulse phase (x-axis of the figure) of 180°.

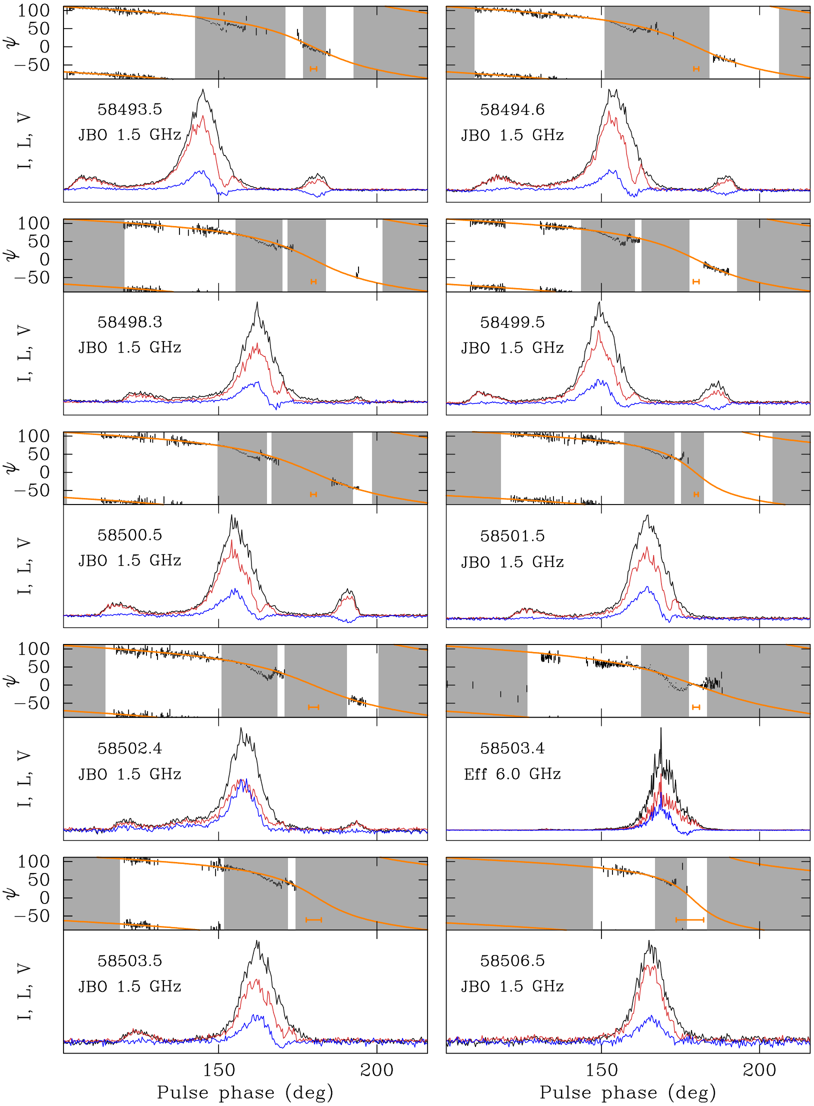

Supplementary Figure 4: Polarised pulse profiles of XTE J1810−197 recorded between MJDs 58493.5 and 58506.5. Top panel: the black error bars represent the PA data while the grey background show the phase ranges where PA data have been excluded from the fit. The uncertainty in the PA is calculated given L and the off-pulse standard deviation of I [1]. The oranges curves show the RVM prediction from the maximum likelihood parameters. The orange horizontal error bar at phase 180° indicates the 95% confidence level on the one-dimensional marginalised posterior of ϕ_0, the position of the RVM inflection point. Bottom panel: The black, red and blue lines show the total intensity, linear and circular polarisation, respectively. The MJD is indicated on the left of the pulse with the telescope and central observing frequency. The pulse profiles are aligned such that the inflection point of the RVM (i.e. $\phi_0 = 0°$) from the free precession analysis is set at the pulse phase (x-axis of the figure) of 180°.

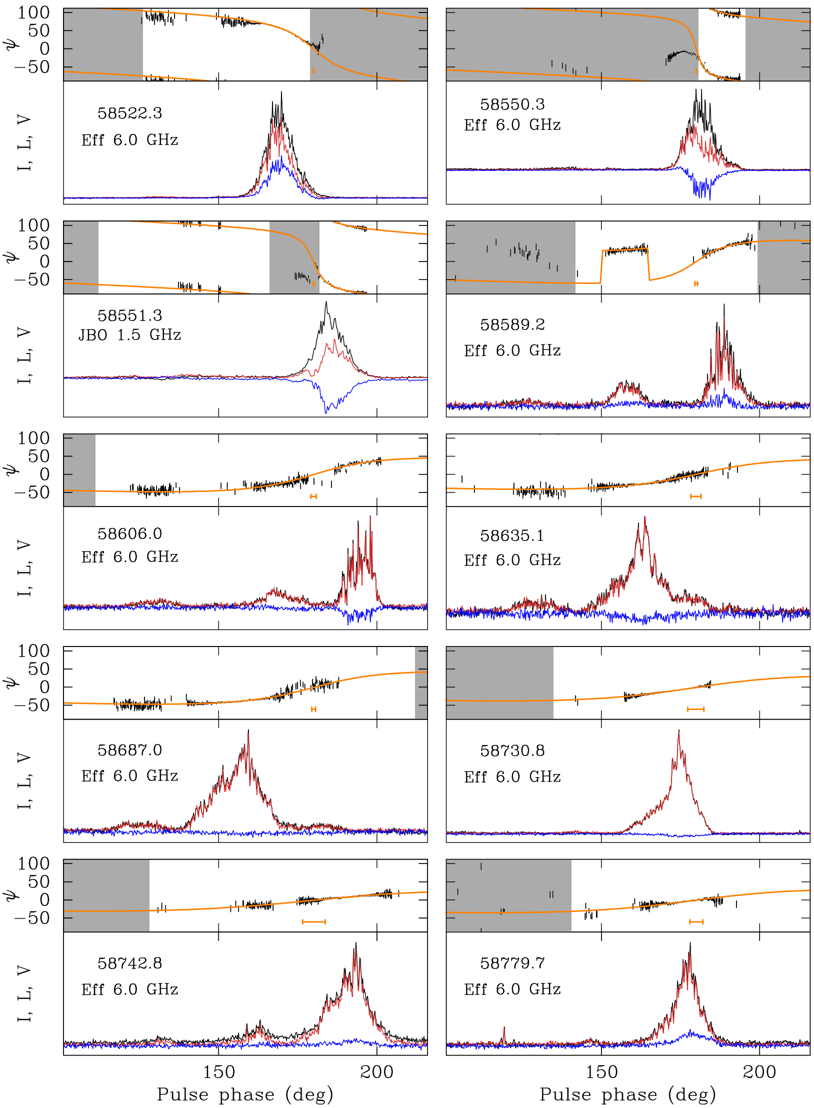

Supplementary Figure 5: Polarised pulse profiles of XTE J1810−197 recorded between MJDs 58522.3 and 58779.7. Top panel: the black error bars represent the PA data while the grey background show the phase ranges where PA data have been excluded from the fit. The uncertainty in the PA is calculated given L and the off-pulse standard deviation of I [1]. The oranges curves show the RVM prediction from the maximum likelihood parameters. The orange horizontal error bar at phase 180° indicates the 95% confidence level on the one-dimensional marginalised posterior of ϕ_0, the position of the RVM inflection point. Bottom panel: The black, red and blue lines show the total intensity, linear and circular polarisation, respectively. The MJD is indicated on the left of the pulse with the telescope and central observing frequency. The pulse profiles are aligned such that the inflection point of the RVM (i.e. $\phi_0 = 0°$) from the free precession analysis is set at the pulse phase (x-axis of the figure) of 180°.

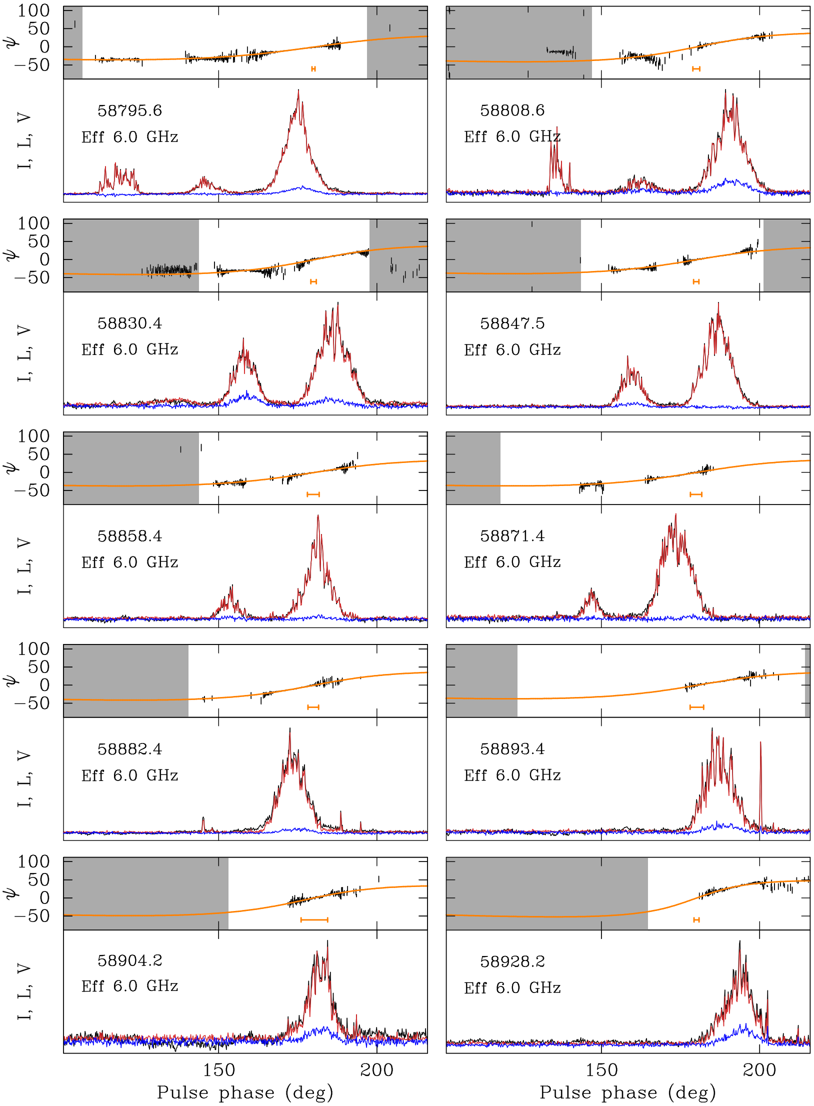

Supplementary Figure 6: Polarised pulse profiles of XTE J1810−197 recorded between MJDs 58795.6 and 58928.2. Top panel: the black error bars represent the PA data while the grey background show the phase ranges where PA data have been excluded from the fit. The uncertainty in the PA is calculated given L and the off-pulse standard deviation of I [1]. The oranges curves show the RVM prediction from the maximum likelihood parameters. The orange horizontal error bar at phase 180° indicates the 95% confidence level on the one-dimensional marginalised posterior of ϕ_0, the position of the RVM inflection point. Bottom panel: The black, red and blue lines show the total intensity, linear and circular polarisation, respectively. The MJD is indicated on the left of the pulse with the telescope and central observing frequency. The pulse profiles are aligned such that the inflection point of the RVM (i.e. $\phi_0 = 0°$) from the free precession analysis is set at the pulse phase (x-axis of the figure) of 180°.

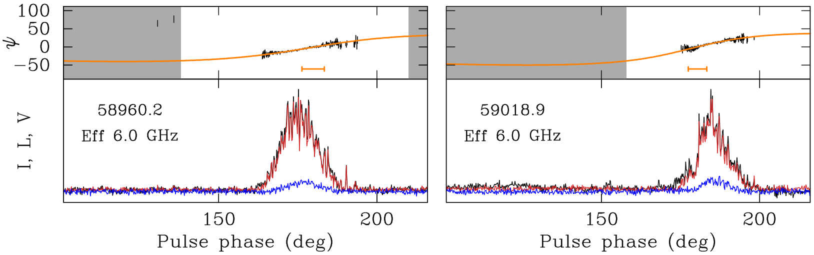

Supplementary Figure 7: Polarised pulse profiles of XTE J1810−197 recorded between MJDs 58960.2 and 59018.9. Top panel: the black error bars represent the PA data while the grey background show the phase ranges where PA data have been excluded from the fit. The uncertainty in the PA is calculated given L and the off-pulse standard deviation of I [1]. The oranges curves show the RVM prediction from the maximum likelihood parameters. The orange horizontal error bar at phase 180° indicates the 95% confidence level on the one-dimensional marginalised posterior of ϕ_0, the position of the RVM inflection point. Bottom panel: The black, red and blue lines show the total intensity, linear and circular polarisation, respectively. The MJD is indicated on the left of the pulse with the telescope and central observing frequency. The pulse profiles are aligned such that the inflection point of the RVM (i.e. $\phi_0 = 0°$) from the free precession analysis is set at the pulse phase (x-axis of the figure) of 180°.

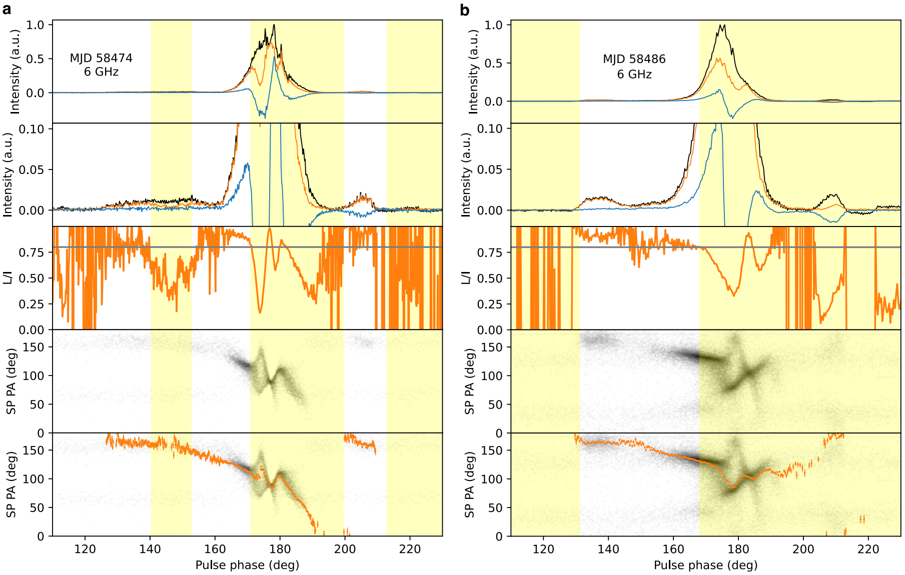

Supplementary Figure 8: Polarimetric pulse profiles at C/X band for two different epochs. Profiles from panels (a) and (b) were recorded on MJDs 58474 and 58486, respectively. For each epoch, the top two panels show the total intensity, linear and circular polarisation with black, red and blue lines, respectively. The MJD and central frequency of the observation is indicated on the left of each pulse profile in the top panels. The second panel from the top is a zoomed-in plot on the low-intensity part of the pulse profile. The third panel from the top shows the fractional linear polarisation of the pulse with the 80% threshold used for the exclusion of the phase ranges. The two bottom panels are the 2-D histograms of the PA values of the single pulses with the average PA value shown in red in the bottom plot. For MJD 58474 and 58486, the histogram are made from 265 and 920 single pulses, respectively. From this plot, we can notice that the dips in linear polarisation correspond to an apparent branching of the PA.

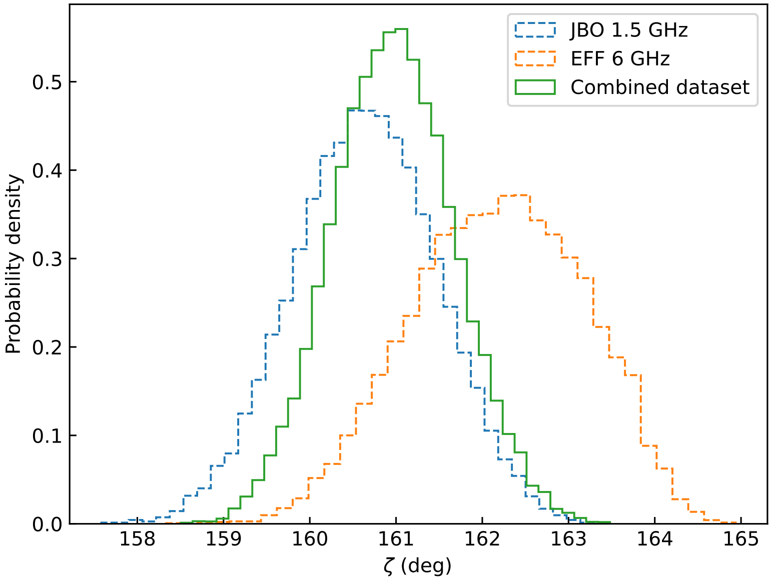

Supplementary Figure 9: Posterior distribution function (PDF) for the ζ parameter in the free precession model. The PDF shown in blue and orange dashed lines represent the results from the separate analysis of the JBO and Effelsberg C/X band data, respectively. The narrower PDF drawn with a green line shows the results from the combined analysis of the JBO and Effelsberg data with $\zeta = 161.0^{\circ} \pm 0.7^{\circ}$

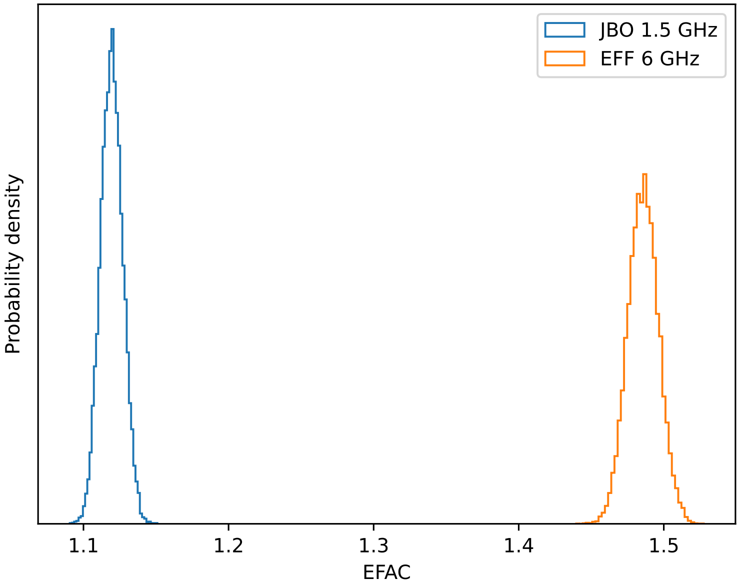

Supplementary Figure 10: Posterior distributions for the two EFAC parameters included in the free precession modelling of the combined dataset. The blue and orange lines represent the posteriors for the EFAC parameters applied to the JBO and Effelsberg C/X band data, respectively.

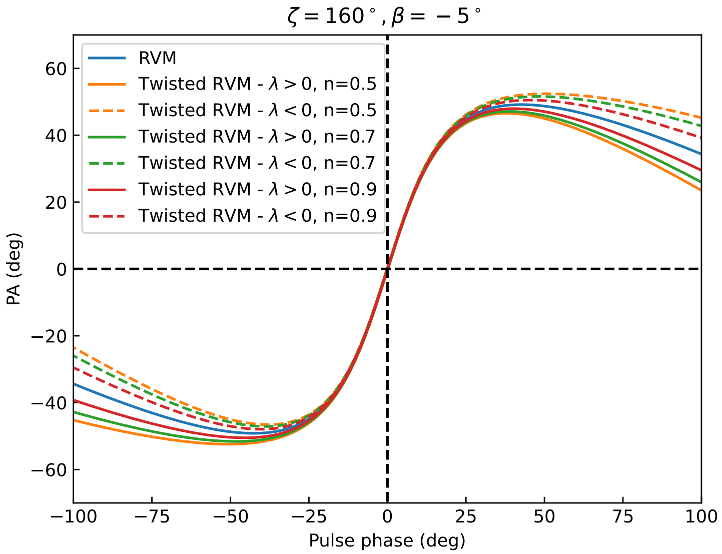

Supplementary Figure 11: Predicted PA for the RVM and the twisted RVM assuming different values for the twist parameter n and its direction. We used the same dipole geometry in all cases, i.e. $\zeta = 160^\circ$ and $\beta = -5^\circ$. The solid and dashed lines of the same color indicate a westward ($\lambda < 0$) and eastward ($\lambda > 0$) twist of the magnetic field lines, respectively. The predicted PAs differ significantly only when the pulse phase is offset from the RVM inflection point at the fiducial pulse phase of 0°.

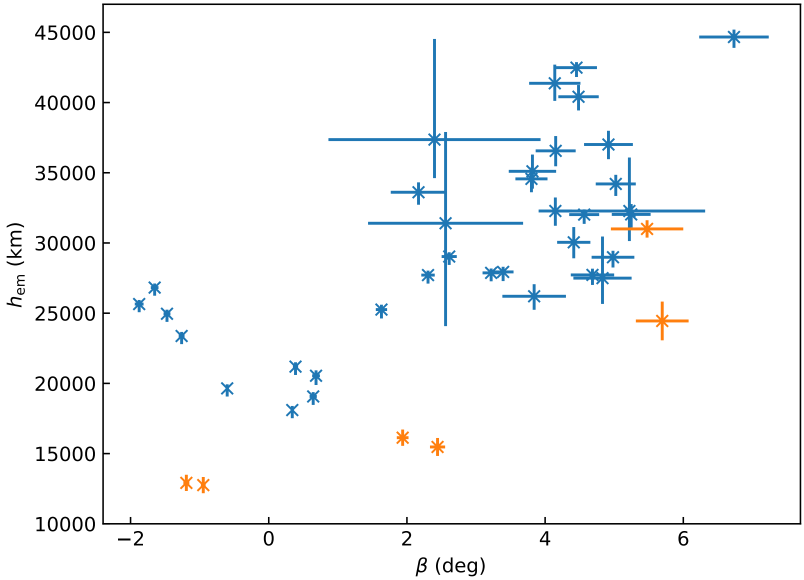

Supplementary Figure 12: Emission height $h_{\rm em}$ as a function of the impact parameter β. The JBO data recorded at 1.5 GHz and Effelsberg data recorded at 6 GHz are represented with blue and orange colors, respectively. According to Eq. 4, the uncertainty in $h_{\rm em}$ comes from the uncertainty in $\phi_{\rm A/R}$ as the uncertainty in $R_{\rm LC}$ is negligible here. To compute the uncertainty in $\phi_{\rm A/R}$, we added in quadrature the uncertainty on the center of the pulse profile with the uncertainty in ϕ_0. We assumed an uncertainty of one phase bin (with 1024 phase bins across the pulse profile) for the center of the pulse and we took the 95% confidence levels on the one-dimensional marginalised posterior for ϕ_0 from the free precession analysis. Similarly to Fig. 2, we also took the 95% confidence levels on the one-dimensional marginalised posterior of β to plot the uncertainty in the abscissa.

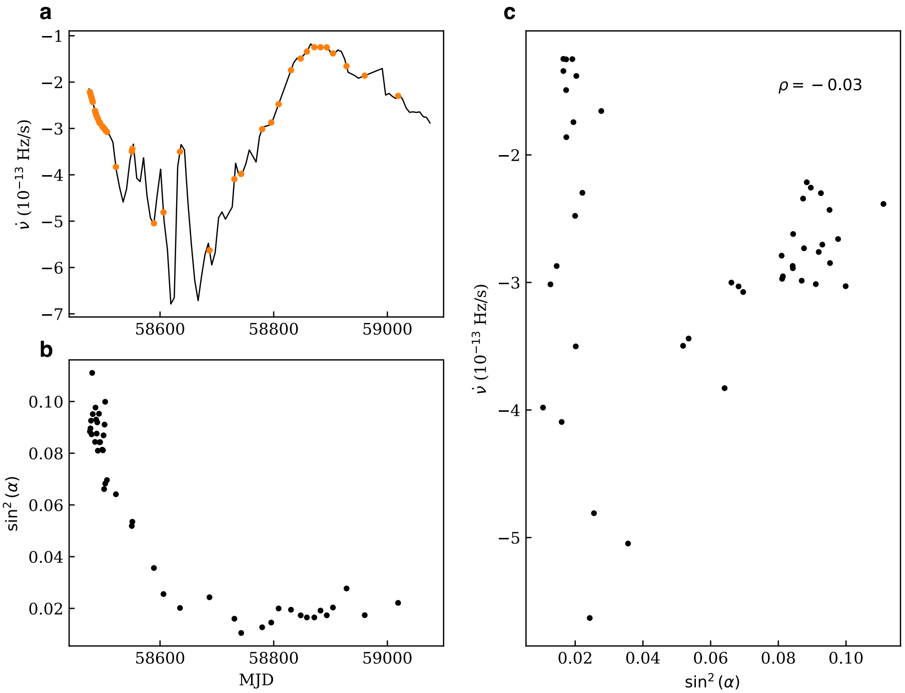

Supplementary Figure 13: Correlation analysis between the magnetar spin-down and its geometry. Panel (a) shows the spin-down $\dot{\nu}$ measured by [2] with the orange points representing the linearly-interpolated $\dot{\nu}$ values at the time of our observations. Panel (b) shows $\sin^2 \alpha$ as derived from our free precession analysis. A Spearman correlation analysis between the magnetar spin-down and $\sin^2 \alpha$ (panel (c)) gives a correlation coefficient $\rho = -0.03$ with a p-value of 0.86.

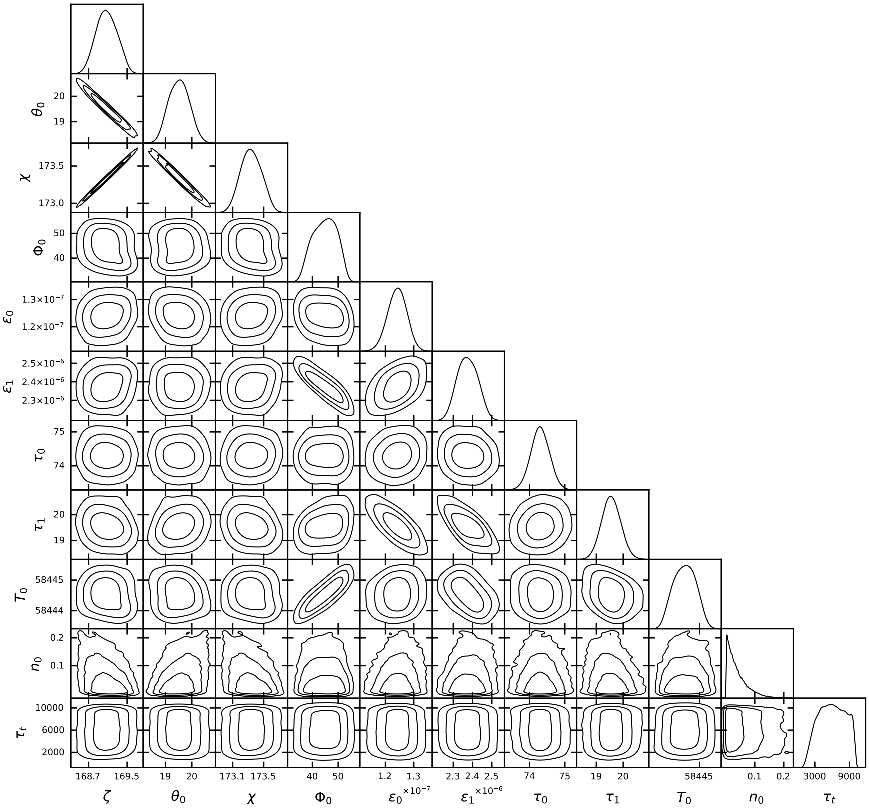

Supplementary Figure 14: One and two-dimensional marginalised posterior distribution for the parameters of the phenomenological model of wobble angle decay with relaxing ellipticity to a non-zero value. In the two-dimensional plots, the three lines represent the 68%,95% and 99.7% confidence levels

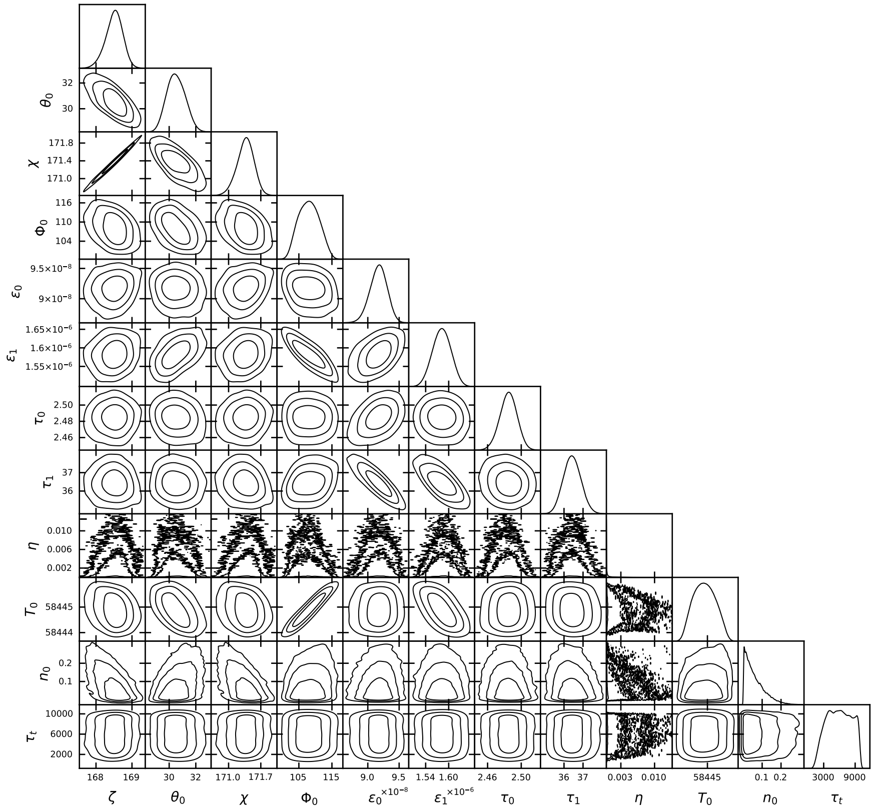

Supplementary Figure 15: One and two-dimensional marginalised posterior distribution for the parameters of the frictional crust-core coupling model. In the two-dimensional plots, the three lines represent the 68%,95% and 99.7% confidence levels

Supplementary Table 1: Model comparison between free and forced precession for the different datasets. The columns indicate the number of epochs included in each dataset (N_{epochs}), the number of EFAC parameters included in the analysis (N_{EFAC}), the corresponding number of dimensions in the nested sampling analysis (N_{dim}) and the log evidence ($\log \mathcal{Z}$).

Dataset	N_{epochs}	N_{EFAC}	N_{dim}	Free precession ($\log \mathcal{Z}$)	Forced precession ($\log \mathcal{Z}$)
JBO L-band	36	1	110	69042.4(6)	68989.0(6)
Eff C/X-band	26	1	80	53519.5(5)	53510.5(5)
Combined dataset	62	2	189	122582.9(6)	122508.3(6)

Supplementary Table 2: Comparison between free precession and forced precession on the combined dataset, including the effects of a relaxing twisted magnetic field. The log evidence value marked in bold indicates the preferred model of free precession that includes an eastward relaxing magnetic field.

Model	Without twist		With twist relaxation		
				$\lambda < 0$	$\lambda > 0$
	N_{dim}	$\log \mathcal{Z}$	N_{dim}	$\log \mathcal{Z}$	$\log \mathcal{Z}$
Forced precession	189	122508.3(6)	192	122503.2(6)	122660.9(6)
Free precession	189	122582.9 (6)	192	122575.1(6)	**122905.2(6)**

Supplementary Table 3: Results of the RVM applied to the 2006 Effelsberg archival data. The columns indicate the MJD and the measured viewing angle ζ and impact parameter β with the uncertainties reported as the 95% confidence levels on the one-dimensional marginalised posterior.

MJD	ζ (deg)	β (deg)
53926	154^{+20}_{-20}	-13^{+11}_{-12}
53934	153^{+20}_{-20}	-14^{+12}_{-13}
53938	151^{+20}_{-30}	-17^{+15}_{-16}
53944	164^{+14}_{-16}	$-9.1^{+8.4}_{-9.8}$

Supplementary Table 4: Table of priors for the parameters used in the different free precession models. For Gaussian prior, the prior range is written as the mean value with the standard deviation in parenthesis. The log-uniform priors are reported here in the $\log_{10}$ space.

Parameter	Type of prior	Prior range
Viewing angle, ζ (deg)	Gaussian	160 (5)
Initial wobble angle, θ_0 (deg)	Uniform	[1;90]
Angle between magnetic and symmetry axis, χ (deg)	Uniform	[0;180]
Initial phase of the precession, Φ_0 (deg)	Uniform	[0;360]
Constant ellipticity of the NS, ϵ_0	Log-uniform	[-9;-6]
Initial ellipticity of the NS, ϵ_1	Log-uniform	[-8;-5]
Frictional coupling time scale, τ_c (s)	Log-uniform	[-1;1]
Wobble angle decay timescale, τ_θ (days)	Log-uniform	[0.5;2.3]
Ellipticity relaxation timescale, τ_ϵ (days)	Log-uniform	[0.5;2.3]
Ratio between the MoI of the crust and the core, η	Log-uniform	[-6;-1]
Start time of precession and outburst, T_0 (MJD)	Gaussian	58445 (1)
Initial twist parameter, n_0	Uniform	[0;1]
Twisted magnetic field relaxation timescale, $\tau_{\rm t}$ (days)	Log-uniform	[1;4]
EFAC	Log-uniform	[-0.4;0.6]

References

[1] Everett, J. E. & Weisberg, J. M. Emission Beam Geometry of Selected Pulsars Derived from Average Pulse Polarization Data. *Astrophys. J.* **553**, 341–357 (2001) .

[2] Caleb, M. *et al.* Radio and X-ray observations of giant pulses from XTE J1810 - 197. *Mon. Not. R. Astron. Soc.* **510** (2), 1996–2010 (2022) .
